# Health service costs and their association with functional impairment among adults receiving integrated mental health care in five low- and middle-income countries: the PRIME cohort study

**DOI:** 10.1093/heapol/czz182

**Published:** 2020-03-09

**Authors:** Dan Chisholm, Emily Garman, Erica Breuer, Abebaw Fekadu, Charlotte Hanlon, Mark Jordans, Tasneem Kathree, Fred Kigozi, Nagendra Luitel, Girmay Medhin, Vaibhav Murhar, Inge Petersen, Sujit D Rathod, Rahul Shidhaye, Joshua Ssebunnya, Vikram Patel, Crick Lund

**Affiliations:** Department of Mental Health and Substance Abuse, World Health Organization, Avenue Appia 20, Geneva 1211, Switzerland; Alan J Flisher Centre for Public Mental Health, Department of Psychiatry and Mental Health, University of Cape Town, 46 Sawkins Road, Rondebosch, 7700 Cape Town, South Africa; Alan J Flisher Centre for Public Mental Health, Department of Psychiatry and Mental Health, University of Cape Town, 46 Sawkins Road, Rondebosch, 7700 Cape Town, South Africa; Centre for Global Mental Health, Health Service and Population Research Department, Institute of Psychiatry, Psychology & Neuroscience, King’s College London, De Crespigny Park, SE5, London, UK; Centre for Global Mental Health, Health Service and Population Research Department, Institute of Psychiatry, Psychology & Neuroscience, King’s College London, De Crespigny Park, SE5, London, UK; Department of Psychiatry, School of Medicine, College of Health Sciences, Addis Ababa University, Arat Kilo, 1176, Addis Ababa, Ethiopia; Centre for Global Mental Health, Health Service and Population Research Department, Institute of Psychiatry, Psychology & Neuroscience, King’s College London, De Crespigny Park, SE5, London, UK; School of Psychology, University of KwaZulu Natal, Umbilo Road, Congella 4013, Durban, South Africa; Butabika Mental Hospital, Plot 2, Butabika Road, P.O. Box 7017, Kampala, Uganda; Transcultural Psychosocial Organization (TPO) Nepal, Baluwatar 44600, Kathmandu, Nepal; Department of Psychiatry, School of Medicine, College of Health Sciences, Addis Ababa University, Arat Kilo, 1176, Addis Ababa, Ethiopia; Sangath, 120, Deepak Society, Chuna Bhatti, Bhopal, Madhya Pradesh 462016, India; School of Psychology, University of KwaZulu Natal, Umbilo Road, Congella 4013, Durban, South Africa; Department of Population Health, London School of Hygiene and Tropical Medicine, Keppel Street, London WC1E 7HT, UK; Centre for Mental Health, Public Health Foundation of India, Unit No. 316, 3rd Floor, Rectangle -1 Building, Plot No. D-4, District Centre Saket, New Delhi-110017, India; Butabika Mental Hospital, Plot 2, Butabika Road, P.O. Box 7017, Kampala, Uganda; Centre for Mental Health, Public Health Foundation of India, Unit No. 316, 3rd Floor, Rectangle -1 Building, Plot No. D-4, District Centre Saket, New Delhi-110017, India; Department of Global Health and Social Medicine, Harvard Medical School, 641 Huntington Avenue, Boston, MA 02115, USA; Alan J Flisher Centre for Public Mental Health, Department of Psychiatry and Mental Health, University of Cape Town, 46 Sawkins Road, Rondebosch, 7700 Cape Town, South Africa; Centre for Global Mental Health, Health Service and Population Research Department, Institute of Psychiatry, Psychology & Neuroscience, King’s College London, De Crespigny Park, SE5, London, UK

**Keywords:** Mental health care, service costs, low- and middle-income countries

## Abstract

This study examines the level and distribution of service costs—and their association with functional impairment at baseline and over time—for persons with mental disorder receiving integrated primary mental health care. The study was conducted over a 12-month follow-up period in five low- and middle-income countries participating in the Programme for Improving Mental health carE study (Ethiopia, India, Nepal, South Africa and Uganda). Data were drawn from a multi-country intervention cohort study, made up of adults identified by primary care providers as having alcohol use disorders, depression, psychosis and, in the three low-income countries, epilepsy. Health service, travel and time costs, including any out-of-pocket (OOP) expenditures by households, were calculated (in US dollars for the year 2015) and assessed at baseline as well as prospectively using linear regression for their association with functional impairment. Cohort samples were characterized by low levels of educational attainment (Ethiopia and Uganda) and/or high levels of unemployment (Nepal, South Africa and Uganda). Total health service costs per case for the 3 months preceding baseline assessment averaged more than US$20 in South Africa, $10 in Nepal and US$3–7 in Ethiopia, India and Uganda; OOP expenditures ranged from $2 per case in India to $16 in Ethiopia. Higher service costs and OOP expenditure were found to be associated with greater functional impairment in all five sites, but differences only reached statistical significance in Ethiopia and India for service costs and India and Uganda for OOP expenditure. At the 12-month assessment, following initiation of treatment, service costs and OOP expenditure were found to be lower in Ethiopia, South Africa and Uganda, but higher in India and Nepal. There was a pattern of greater reduction in service costs and OOP spending for those whose functional status had improved in all five sites, but this was only statistically significant in Nepal.

**Key Messages**

- Evaluation of the costs of service uptake represents an important but rarely studied element of mental health service scale-up in low- and middle-income countries.
- This study assessed the costs and out-of-pocket (OOP) payments of persons with mental disorder receiving integrated primary mental health care in five countries.
- OOP expenditures were higher at baseline for persons with greater functional impairment, while at 1-year follow-up there was a trend for greater cost reductions among those whose functional capacity had improved.

## Introduction

Following agreement on a global mental health plan of action (Lancet global mental health group, 2007; Collins *et al.*, 2011; WHO, 2013), increasing effort has been made to scale-up services for mental, neurological and substance use (MNS) disorders in low- and middle-income countries (LMICs) through integration of mental health into primary health care (PHC). Alongside and complementary to these mental health policy developments, a strong consensus has formed around universal health coverage (UHC) as a central aim of health systems and sustainable development (WHO, 2010a; United Nations, 2015). The essential implication of these global policy goals is that people with MNS disorders should be able to access effective and affordable health services. Regarding affordability, the potentially high or impoverishing cost to households of paying for the health services and goods they need is a fundamental concern of ongoing UHC reforms. Direct out-of-pocket (OOP) payments for health services and goods represent a regressive form of health financing—penalizing those least able to afford care—and represent a channel through which impoverishment may occur or deepen (Chisholm *et al.*, 2015).

In this context, assessment of OOP payments made by households, as well as the cost of service provision by local health authorities, represents an integral component of mental health service scale-up evaluation. In this study, the amount—as well as distribution across different payers—of service costs and OOP expenditures that are associated with mental health service uptake were assessed, both prior to and following its integration into PHC at the district level in five LMICs in sub-Saharan Africa and South Asia.

Investment in mental health service scale-up is expected to lead to improved health, functioning and well-being for people with MNS disorders (Simon *et al.*, 2002; Srinivasa Murthy *et al.*, 2005; Lund *et al.*, 2012; De Silva *et al.*, 2013; Asher *et al.*, 2017); accordingly, the relationship over time between incurred service costs and assessed levels of functional impairment was examined. Analysis of the relationship between costs and functional status or outcome was structured around two hypotheses:

1. Among adults with mental disorders in participating districts, health service costs and OOP expenditures incurred over the previous 3 months are positively associated with functional impairment;
2. Among adults with mental disorders in participating districts, total health service costs and OOP expenditures incurred 3–6 and 12 months after mental health treatment initiation are reduced, and greater functional improvement is associated with lower service costs.

## Methods

### Study setting and design

Analysis of resource needs, costs and outcomes was carried out for study participants recruited and followed up as part of an intervention cohort study carried out in five districts in sub-Saharan Africa and South Asia participating in the Programme for Improving Mental health carE (PRIME) study (Lund *et al.*, 2012). The overall aim of the PRIME study was to generate evidence on the implementation and scaling up of integrated packages of care for priority mental disorders in primary and maternal health care settings in Ethiopia, India, Nepal, South Africa and Uganda. The primary basis for the mental health care intervention package was the WHO mhGAP intervention guide, which provides evidence-based clinical decision-making algorithms for a set of priority MNS disorders for use in non-specialized health settings (WHO, 2010b). Key treatment and care inputs included basic psychosocial treatment—and where indicated and available, more intensive psychotherapy—by trained general health care workers, and pharmacological treatment with essential psychotropic medications. In South Africa, chronic care guidelines for Adult Primary Care that include mental health care were the main basis for treatment.

A phased, multi-methods approach was employed across all participating sites for the development, implementation and evaluation of the care packages, including formative research with local stakeholders on the design of a mental health care plan; case studies of district-level mental health systems; studies of cohorts of individuals treated through the mental health care plans; facility-based surveys to assess changes in case detection; and community-based surveys to assess changes in coverage and stigma (Lund *et al.*, 2012; De Silva *et al.*, 2016). The specific objective of the PRIME cohort study, which is the focus for this analysis, was to assess the changes in social, health and economic outcomes over 12 months for people identified with a range of prioritized mental health conditions [depression, alcohol use disorders (AUD), psychosis and epilepsy] who had initiated primary care-based mental health care (De Silva *et al.*, 2016; Baron *et al.*, 2018). The cohort study was observational and naturalistic in design, rather than controlled and experimental, so the primary interest was in testing associations over time, in this case, between resource costs and functional outcomes.

Study settings spanned a diverse range of socio-cultural, urban/rural and economic contexts, including extremely under-resourced settings (Ethiopia, Uganda), a fragile state setting (Nepal) and middle-income countries marked by high levels of socio-economic inequality (India and South Africa). Further details about the socio-economic and health service context in these diverse settings can be found elsewhere (Lund *et al.*, 2012; Hanlon *et al.*, 2014). Reflecting this diversity of local needs, priorities and realities, mental health care plans differed somewhat with respect to selected conditions, treatment modalities or medications used, and types of health workers trained, while working towards a common goal with the same methodological approach.

The sample sizes for the country cohorts were guided by data regarding the mean expected change in symptom severity scores between baseline and end-line as a result of receiving evidence-based treatments; calculations showed that a meaningful clinical impact for treatment of depression and alcohol use disorder and could be detected with >200 participants per disorder per country and for treatment of psychosis and epilepsy with >150 participants per disorder per country (Baron *et al.*, 2018). Each cohort study commenced as each district mental health care plan had been embedded (Fekadu *et al.*, 2016; Jordans *et al.*, 2016; Kigozi *et al.*, 2016; Petersen *et al.*, 2016; Shidhaye *et al.*, 2016).

Prospective participants in the cohort study consisted of adults identified by trained primary care providers (as per the respective country mental health care plan) as having one of four locally prioritized MNS disorders: psychosis (predominantly schizophrenia and some cases of bipolar disorder in some of the sites), AUD and, in Ethiopia, Nepal and Uganda, epilepsy. India and South Africa did not select epilepsy as a priority disorder in their district mental health care plans, while South Africa and Uganda did not include AUD. Participants meeting diagnostic criteria or screening positive for more than one condition were allocated to a primary disorder based on pre-assigned rules; e.g. priority was given to alcohol use disorder in cases where participants screened positive for depression as well as AUD, while priority was given to psychosis in the event of dual diagnosis with AUD or depression. Inclusion in the study was conditional on informed consent as well as ability to speak in the local language and complete the study questionnaire (for non-literate cases of psychosis in Ethiopia, verbal consent was accompanied by a fingerprint in the presence of a literate witness, while for those who lacked capacity to consent but were not refusing participation, caregiver permission was obtained). Consecutive sampling of diagnosed patients from primary care clinics was carried out until target sample sizes were reached. Cohort participants were informed about and offered treatment for their MNS disorder by trained PHC providers, and were assessed by the local research team 3 or 6 months (mid-line visit) and 12 months (end-line visit) after the baseline assessment. The mid-line visit was set to coincide with the time point at which the full effect of treatment is expected to occur: for depression and AUD, this was 3 months (±2 weeks) after baseline; for psychosis and epilepsy, it was at 6 months (±2 weeks).

Approval for the PRIME cohort study was obtained from the local ethics boards of participating countries, as well as from the authors’ institutions.

### Data collection

A trained interviewer orally administered a structured questionnaire at all three study visits at the participants’ home or local clinic. The respondent was the participant, aside from the psychosis cohorts in Nepal and Ethiopia where caregivers responded on behalf of the participant (for all cases in Nepal and for those lacking capacity in Ethiopia). The cohort questionnaire had sections pertaining to the participants’ sociodemographic characteristics, symptom severity, suicidal ideation, functioning and health service utilization (Baron *et al.*, 2018). The service utilization section had items about all inpatient admissions in the past year and outpatient visits in the preceding 3 months, including the reason for the admission or visit, the number and average duration of visits and any privately incurred expenditure. Participants were also asked about what if any pharmacological or psychosocial treatment they had received or taken in the previous 3 months. The measure of functional impairment was the 12-item version of the WHO Disability Assessment Schedule (WHO-DAS 2; http://www.who.int/classifications/icf/more_whodas/en, accessed 12 August 2019). Further details relating to the design, recruitment and data collection procedures for the PRIME cohort study are available via a published protocol paper (Baron *et al.*, 2018).

### Data preparation

For assessment of the relative severity of functional impairment, we derived a summary score from the WHO-DAS 2 using the polytomous scoring algorithm (Ustun *et al.*, 2010) and then dichotomized cases into those with higher vs lower level of impairment, using the 85th percentile as the cut-off level for allocating cases. This cut-off level is in line with a reported disability prevalence of 15% in the adult population based on a global analysis of the World Health Survey dataset (WHO, 2011).

For assessment of costs, for which a health systems perspective was used, several dimensions were of interest as shown in Scheme 1 below:

1. total health service costs (for mental health care and other services too), including psychotropic medication (expenditures incurred by households vs government or non-state actors were separately identified and measured to enable an analysis of funding source for these costs); this quantum is denoted A3 in the scheme;
2. total travel and time costs, including both financial payments made by individuals or households for transportation as well as the estimated economic value of time spent accessing services; this quantum is denoted B3 in the scheme;
3. total OOP costs, made up of all the financial payments made by individuals or household members on travel, consultation fees and medicines; this quantum is denoted C1 in the scheme.

For health service costs, data from the cohort questionnaire on the use of services and any medication at baseline, mid-line and end-line assessments were converted into monetary values by multiplying reported quantities of service use by locally applicable prices (e.g. for drugs) or country-specific unit cost estimates (e.g. inpatient day or outpatient visit). Unit costs varied according to the sector in which services are provided (public vs private sector). For all countries other than South Africa, unit costs of health services are based on WHO-CHOICE estimates, updated to the year 2015 (https://www.who.int/choice/country/country_specific/en/, accessed 19 August 2019) and medication prices were taken from the International Medical Products Price Guide (http://mshpriceguide.org/en/home/, accessed 19 August 2019). Derived values were shared and checked with local team investigators and agreed to be used as the basis for service costs. For South Africa, unit costs of health services are taken from the Department of Health’s Uniform patient fee schedule, and drug prices are taken from the Medicine Price Registry Database. The full set of unit costs used in the analysis is provided in Supplementary Appendix S1. Health service costs were categorized into medication, inpatient care and outpatient care, with the latter further split into mental health services, general health services and indigenous or traditional services. For travel and time costs, any privately incurred and reported costs associated with travel to and from health care facilities such as bus fares were included, together with the estimated monetary value of time spent accessing and waiting for care [derived by multiplying recorded time spent (in minutes) by the average wage rate per minute reported by local site participants].

**Table:** Scheme 1 Categorization of costs

	Health service costs	Travel and time costs	OOP costs
Financial costs			
1. Households	A1. OOP spending (fees, consultations)	B1. OOP spending (transport expenses)	C1. **Total household OOP costs** (A1 + B1)
2. Government or non-state actors	A2. Health service provision / expenditure	Not applicable	Not assessed
Total	A3. **Total health service costs**	Not applicable	Not applicable
Non-financial costs			
1. Households	Not applicable	B2. Value of time spent accessing / waiting for services	Not applicable
2. Government or non-state actors	Not applicable	Not applicable	Not applicable
Total	Not applicable	B3. **Total travel and time costs**	Not applicable

All reported costs are in US dollars for the year 2015 (the year in which data collection commenced), using the average exchange rate. No adjustment was made for purchasing power parity between the participating countries since the focus of interest was on the actual resource costs incurred in each country (rather than a comparison between them, whereby differences in the relative price of goods and services would need to be taken into account). Comparison of costs at the different time points was for the 3-month period preceding them. Annual costs were approximated by reference to mid-line and end-line assessments (e.g. doubling of 6- and 12-month estimates for psychosis and epilepsy).

Cost data for a small number of individuals were implausibly large (e.g. greater than the possible product of outpatient visits in the previous 3 months); nine cases were removed from the dataset on this basis. Participants with missing data for an entire section were excluded from analysis, while participants with individual items missing had country-specific mean values imputed. Data tables with and without adjustments for extreme outliers and missing data were generated and compared for their effect on results for the reference case (extreme outliers removed, with imputation for specific variables only, not whole sections).

### Statistical analysis

To assess the first hypothesis that health services and OOPs costs are positively associated with functional impairment at baseline, multivariate linear (ordinary least squares) regression analyses were conducted on baseline cost estimates of (1) total health service cost and (2) total OOP cost (as the dependent variables) and functional impairment as the independent variable, adjusting for disorder.

To assess the second hypothesis, that health service costs and OOP expenditures decrease over time and are associated with greater improvement in functioning, linear regression was performed with the change in costs at end-line assessment as dependent variable, and increase or decrease in functional impairment as categorical independent variables. Models were controlled for baseline costs and disorder.

Data analysis was first carried out for the entire cohort sample in each site (using functional impairment alone as the measure of comparison), followed by analysis disaggregated by specific disorder, including a break-down of incurred cost into different service components (general health care, mental health care, indigenous health care). Given the skewed distribution of the dependent variables, a non-parametric bootstrap with 1000 repetitions was implemented for estimating the coefficients, 95% confidence intervals (CIs) and *P*-values in all regression analyses.

## Results

### Sociodemographic characteristics of the cohort sample

Across the districts of the five participating countries, a total of 2206 cases were recruited into the cohort study. Following removal of cost outliers (9 cases) and cases with missing sections of resource use data (182 cases), the total sample for this analysis was 2015 cases. Of the total sample, 12 (0.7%) and 27 participants (1.6%) had missing sections of resource use data at mid-line and end-line assessments, respectively, and so total health service costs could not be calculated. Sample sizes per country varied from 245 in South Africa to 540 in Ethiopia. Included cases were evenly distributed between the different disorders of depression (33%), alcohol use disorders (20%), psychosis (24%) and epilepsy (23%), but as shown in Supplementary Table S1, there were marked differences in case distribution at the country level; e.g. in line with their mental health care plans, there was no cohort sample for epilepsy in India and South Africa and no cohort sample for alcohol use disorders in South Africa and Uganda.

As shown in Table 1, women made up the majority of the analysed sample in Uganda (55%) and South Africa (75%), while in the other sites there were more men (55–65% of cases). Samples varied widely in terms of marital status, with <30% having a partner in Uganda compared with >80% in Nepal and India. Regarding educational levels, 21–25% of cases had completed primary school education or above in Ethiopia and Uganda, compared with around 50% in India and Nepal and 68% in South Africa. Looking across the socio-economic characteristics of the country samples, they are either marked by low levels of educational attainment (Ethiopia and Uganda) or high levels of unemployment (Nepal, South Africa and Uganda).

**Table 1:** Sociodemographic characteristics of the PRIME cohort participants, 2015–17

	Ethiopia (*n* = 540)		India (*n* = 483)^a^		Nepal (*n* = 433)		South Africa (*n* = 245)		Uganda (*n* = 295)	
	*n*	%	*n*	%	*n*	%	*n*	%	*n*	%
Sex										
Male	327	60.6	315	65.2	237	54.7	61	24.9	132	44.8
Female	213	39.4	168	34.8	196	45.3	184	75.1	163	55.2
Age (years)										
16–25	175	32.4	88	18.2	40	9.2	28	11.4	124	42.0
26–35	148	27.4	135	28.0	116	26.8	58	23.7	88	29.8
36–50	155	28.7	185	38.3	172	39.7	70	28.6	65	22.0
≥51	62	11.5	75	15.5	105	24.3	89	36.3	18	6.1
Marital status										
No partner	307	56.9	42	8.7	78	18.0	134	54.7	213	72.2
Has a partner	233	43.1	441	91.3	355	82.0	111	45.3	82	27.8
Educational level										
Uneducated/illiterate	303	56.3	140	29.0	105	24.3	9	3.7	60	20.3
Non-formal/less than primary	101	18.8	115	23.8	110	25.4	69	28.2	172	58.3
Primary school and above	134	24.9	228	47.2	218	50.3	167	68.2	63	21.4
Employment^b^										
Unemployed/not salaried	2	6.9	152	31.5	217	50.1	185	75.5	196	66.4
Employed	27	93.1	331	68.5	216	49.9	60	24.5	99	33.6
Food insecurity^b^										
No	29	96.7	460	95.4	102	23.6	137	55.9	233	79.0
Yes	1	3.3	22	4.6	331	76.4	108	44.1	62	21.0

a Sociodemographic data missing for 19 cases.

b In Ethiopia, baseline data collected only for AUD cohort.

### Description of health service costs at baseline assessment

The upper part of Table 2 summarizes key categories of cost incurred by the sampled populations (all diagnoses combined), including health care services, travel and time costs and OOP expenditures. Total estimated health service costs per case for the 3 months preceding baseline assessment (including services or goods paid for privately by households as well as by government or non-state actors) amounted to over US$20 in South Africa, $10 in Nepal and US$3–7 in the remaining three country settings. The largest contribution to service costs was outpatient care, followed by inpatient care and then medications. Costs associated with accessing services show that, in some but not all countries, these added significantly to the overall economic impact of ill-health; in Ethiopia, e.g. travel costs amounted to double the cost of service provision itself (an average of US$11 per case over 3 months). Total OOP expenditures related to travel, medication and consultations over the previous 3 months ranged from US$2 in India to US$10–16 in the low-income country contexts of Ethiopia and Nepal.

**Table 2:** Baseline costs of health care, travel and time for PRIME cohort participants (in US dollars for the year 2015)

		Ethiopia			India			Nepal			South Africa			Uganda		
		*n*	Mean	SD	*n*	Mean	SD	*n*	Mean	SD	*n*	Mean	SD	*n*	Mean	SD
Service cost, by type of care/service^a^		540	5.72	21.08	502	3.00	9.06	433	10.17	29.95	245	23.01	41.22	295	6.70	10.86
Inpatient care		540	1.47	10.26	502	1.24	8.78	433	3.89	21.43	245	7.67	32.68	295	1.80	7.14
Outpatient care		540	4.25	17.86	502	1.35	1.67	433	4.49	20.12	245	13.37	19.08	295	3.25	7.83
Mental health services		540	0.69	2.93	502	0.33	1.05	433	1.10	15.64	245	2.02	10.21	295	1.02	2.43
General health services		540	1.95	11.57	502	0.85	1.13	433	1.62	5.87	245	10.02	12.34	295	0.95	3.05
Indigenous/traditional services		540	1.61	13.01	502	0.17	0.62	433	1.27	11.14	245	1.33	6.08	295	1.29	6.87
Medication					502	0.41	0.57	433	1.79	2.56	245	1.97	7.46	295	1.64	2.11
Travel time and costs^a^																
Accessing/waiting for services		540	2.27	5.20	502	0.98	1.33	433	1.84	4.37	245	12.63	14.90	295	0.98	1.64
Travel payments		540	11.16	52.31	502	0.59	1.04	433	0.64	2.62	245	1.18	4.24	295	1.74	4.53
Total out-of-pocket expenditure by households^a^ (travel costs, fees and medication)		540	15.78	61.64	502	2.26	6.96	433	10.43	30.59	245	6.68	13.12	295	5.63	10.17
Service cost, by level of functional impairment																
Lowest (<85th percentile)		170	3.96	17.32	309	1.69	3.36	233	9.08	29.02	108	20.90	41.01	125	5.15	3.76
Higher (≥85th percentile)		367	6.56	22.67	173	4.77	13.83	200	11.44	31.03	137	24.67	41.45	170	7.83	13.33
Difference (95% CI)	Higher impairment	2.40 (0.13–4.67)*			3.09 (0.76–5.42) **			2.16 (−3.88 to 8.20)			6.60 (−3.54 to 16.74)			2.36 (−0.09 to 4.82)^m^		
OOP expenditure, by level of functional impairment																
Lowest (<85th percentile)		170	14.24	77.07	309	1.33	2.86	233	9.03	29.36	108	3.91	12.07	125	4.05	4.61
Higher (≥85th percentile)		367	16.52	53.43	173	3.41	10.21	200	12.05	31.96	137	6.50	13.94	170	6.79	12.69
Difference (95% CI)	Higher impairment	0.04 (−11.95 to 12.03)			2.08 (0.37–3.79)*			2.78 (−2.93 to 8.50)			0.45 (−2.86 to 3.80)			2.69 (0.38–5.01)*		

a All cost estimates relate to the 3-month period leading up to baseline assessment.

* *P* < 0.05 ;

** *P* < 0.01;

m, marginal.

SD, standard deviation.

When disaggregated by disorder, cases of psychosis incurred a relatively high level of service cost, travel time and costs, as well as OOP spending; the only exceptions to this were for service cost in Ethiopia—where alcohol use disorders had slightly higher costs—and for OOP spending in South Africa, where depression had a slightly higher level (Supplementary Table S2).

### Service costs and their association with functional impairment at baseline assessment (hypothesis 1)

The lower part of Table 2 compares overall health service costs and OOP spending at baseline assessment by level of functional impairment. In all sites, higher health service costs were found to be associated with cases with greater functional impairment; there was a statistically significant difference in Ethiopia (cost difference: 2.40; 95% CI 0.13–4.67) and India (3.09; 95% CI 0.76–5.42). Similarly, with respect to OOP spending estimates, higher costs were seen for more functionally impaired cases in all sites; cost differences were statistically significant in India (2.08; 95% CI 0.37–3.79) and Uganda (2.69; 95% CI 0.38–5.01).

Tests of association were also performed at the more disaggregated level of specific disorders (Supplementary Table S2). For psychosis, a positive association between functional impairment and service costs as well as OOP spending was observed in each country; differences were statistically significant in Ethiopia and Uganda. A significant positive association was also observed for depression cases in India. For other countries and disorders, the picture was more mixed and no clear trend could be observed.

### Service costs and their association with functional impairment over time (hypothesis 2)

Total service costs and OOP payments at baseline, mid-line and end-line assessment for the combined samples in each country (with any of the selected mental health conditions) are shown in Table 3, along with changes in costs over time for individuals whose levels of functional impairment were assessed to have improved or worsened. In Ethiopia, and to a lesser extent Uganda, there was an appreciable and significant reduction in service costs and OOP expenditure at both mid-line and end-line assessment. In India and Nepal, by comparison, total service costs and OOP expenditure was modestly higher at end-line assessment than at baseline assessment.

**Table 3:** Health care costs and out-of-pocket payments for PRIME cohort participants at baseline, mid-line and end-line assessments (in US dollars for the year 2015)

	Ethiopia			India			Nepal			South Africa			Uganda		
	*n*	Mean	SD	*n*	Mean	SD	*n*	Mean	SD	*n*	Mean	SD	*n*	Mean	SD
Total service cost in last 3 months															
Baseline	540	5.72	21.08	502	3.00	9.06	433	10.17	29.95	245	23.01	41.22	295	6.70	10.86
Mid-line	367	1.95	6.53	465	3.32	12.26	367	7.24	16.22	194	47.12	66.28	282	5.53	7.33
End-line	446	1.65	6.95	433	5.18	19.44	370	11.19	35.41	183	17.36	30.53	259	3.59	5.21
Test of difference (coefficient, 95% CI)															
Mid-line vs baseline	−4.08 (−5.62 to −2.55)***			0.32 (**−**0.67 to 1.31)			−3.16 (−5.73 to −0.60)**			26.25 (16.26–36.23)***			−1.15 (−2.32 to 0.03)^m^		
End-line–baseline	−3.72 (−5.09 to −2.34)***			2.14 (0.27–4.01)*			0.75 (**−**3.34 to 4.85)			**−**3.56 (**−**9.33 to 2.21)			***−*** 3.03 (−4.11 to −1.95)***		
Change in total service cost (end-line–baseline)															
Functioning worsens^a^	182	−4.20	25.14	209	2.72	21.77	114	6.43	50.30	66	−0.46	53.49	94	−1.28	8.02
Functioning improves^b^	259	−2.64	15.12	203	2.04	21.34	256	−2.24	41.95	117	−4.87	36.52	164	−3.86	13.45
Test of association (*β*, 95% CI)	−0.59 (−2.19 to 1.02)			0.01 (−3.90 to 3.92)			−9.29 (−18.15 to −0.43)*			−8.84 (−18.97 to 1.28)^m^			−0.49 (−1.88 to 0.90)		
Total private, out-of-pocket cost in last 3 months															
Baseline	540	15.78	61.66	502	2.26	6.96	433	10.43	30.59	245	6.68	13.12	295	5.63	10.17
Mid-line	367	4.05	10.14	465	2.64	12.14	367	7.75	17.19	194	5.57	11.81	282	4.87	7.29
End-line	446	3.26	6.96	433	4.73	19.25	370	11.65	36.81	183	4.96	9.17	259	3.95	6.09
Test of difference (coefficient, 95% CI)															
Mid-line–baseline	−12.95 (−17.75 to −8.16)***			0.39 (**−**0.57 to 1.36)			−2.96 (−5.58 to −0.35)*			**−**0.24 (**−**2.12 to 1.65)			**−**0.79 (**−**1.93 to 0.35)		
End-line–baseline	−12.40 (−16.45 to −8.34)***			2.42 (0.52–4.32)*			0.93 (**−**3.30 to 5.16)			**−**1.02 (**−**2.83 to 0.78)			***−*** 1.69 (−2.87 to −0.50)**		
Change in out-of-pocket cost (end-line–baseline)															
Functioning worsens^a^	182	−15.65	86.01	209	2.86	21.70	114	7.03	49.82	66	0.52	15.56	94	−0.34	7.85
Functioning improves^b^	259	−9.94	43.51	203	2.13	19.26	256	−2.24	43.67	117	−1.85	15.07	164	−2.45	13.30
Test of association (*β*, 95% CI)	−1.07 (−2.57 to 0.43)			−0.21 (−4.10 to 3.69)			−9.50 (−18.18 to −0.81)*			−2.96 (−6.08 to 0.17)^m^			−0.09 (−1.58 to 1.41)		

a Defined as a follow-up WHO-DAS score equal to or greater than a WHO-DAS score at baseline.

b Defined as a follow-up WHO-DAS score smaller than a baseline WHO-DAS score.

* *P* < 0.05;

** *P* < 0.01

*** *P* < 0.001;

m, marginal.

SD, standard deviation.

Further tests of association were carried out at the level of specific disorders in each country cohort (Supplementary Table S3a–e), including for different components of service cost (mental health care, general health care and indigenous care). In Ethiopia and Uganda, this analysis revealed a consistent pattern of cost reductions across different service components and disorders. In the other three countries, such disaggregated analysis enabled us to isolate the specific contribution of different service components to total costs over time. In South Africa, e.g. higher costs observed for the depression cohort at mid-line assessment are accounted for by a substantial increase in mental health care costs in the previous 3 months (which then return to baseline levels at end-line assessment). In India, increased utilization of mental health care services over time is the key driver of higher costs and OOP expenditure for the psychosis cohort at end-line assessment, while for the depression cohort the increased cost at end-line assessment is accounted for by greater uptake of general health care. In three out of the four disorder-specific cohorts in Nepal, increased service costs at end-line assessment were attributable to greater uptake of general health care services.

When combined country cohorts were stratified by level of functional improvement, we found modest evidence for the hypothesized inverse association with service costs and OOP expenditure (Table 3). There was a significant cost difference by end-line assessment in favour of those whose functioning had improved in Nepal (coefficient for service cost: −9.29; 95% CI −18.15 to −0.43; coefficient for OOP expenditure: −9.50; 95% CI −18.18 to −0.81), a marginal cost difference in South Africa, and an inverse but not statistically significant association elsewhere. Again, more specific analyses of these associations at the level of specific disorders and service components provided additional context and explanation (Supplementary Table S3a–e); in particular, the hypothesized inverse relationship between functional improvement and service costs as well as OOP expenditure was found to be especially evident for the depression cohort in Nepal (service cost difference: −15.44; 95% CI −30.89 to 0.02; OOP difference: −15.23; 95% CI −30.58 to 0.13) and South Africa (service cost difference: −9.57; 95% CI −19.66 to 0.53; OOP difference: −2.92; 95% CI −5.87 to 0.04).

## Discussion

Alongside consideration of the impact of mental health service scale-up on users’ clinical and functioning outcomes, a further key objective of the PRIME study was to assess the cost, feasibility and affordability of agreed mental health care plans in each of the participating districts (Lund *et al.*, 2012; De Silva *et al.*, 2016). Assessment of the expected costs of scaled-up services in each district had been carried out in an earlier formative phase of the research programme to inform the development of one mental health care plan, based on an epidemiologically informed model of resource need called the mhGAP costing tool (Chisholm *et al*., 2016). Results from the modelling exercise indicated that the annual service cost of delivering a scaled-up package of care to the local population would amount to less than US$1 per capita in all sites except South Africa (Chisholm *et al*., 2016). The predicted cost per treated case of disorder, which underlie such population-level estimates, was also reported; e.g. the annual cost per treated case of depression was estimated at less than US$50 in all sites other than South Africa.

The PRIME cohort study, which was the focus of this analysis, has enabled a new set of cost estimates to be calculated from prospective empirical observation and has shown that actually incurred resource costs closely approximate predicted values for two of the disorders—depression and epilepsy—but were more than predicted for alcohol use disorders and less than predicted for psychosis. In fact, examination of disorder-specific cost estimates in the current study (see Supplementary Appendix S3a–e) shows only modest variation in overall service cost between disorders—a factor of less than two—and for three of the participating country sites (India, Nepal and Uganda) the estimated annual cost per case fell within a range of US$15–50, no matter what the clinical diagnosis was. Estimated annual treatment costs for each disorder in Ethiopia were each less than US$10 (at least two times less than predicted in the earlier modelling exercise), while in South Africa the cost per treated case of depression—the only disorder for which an estimate could be reliably made—was close to US$100, again considerably less than predicted. A key reason for these differences is that the actual availability and use of local services—particularly secondary care—is below the service norms assumed in the modelling exercise.

An important dimension of cost analysis and health financing that could not be easily assessed at the planning stage but well captured in the PRIME cohort study is the contribution made by service users towards the cost of their care. An important finding arising from this analysis is the relatively high level of OOP expenditures at baseline assessment in the low-income settings of Ethiopia, Nepal and to a lesser extent, Uganda (each with a gross national income per capita of less than US$750). In Ethiopia, this financial burden on service users and their households was driven by travel expenses (accessing care) while in the other two sites it was driven by consultation fees and expenditure on medicines (receiving care). Moreover, it was found that, as hypothesized, such OOP expenditures tended to go up with the level of functional impairment, which is likely to further exacerbate inequalities with respect to accessible care for all.

Based on insights from earlier observational studies (Simon *et al.*, 2002; Srinivasa Murthy *et al.*, 2005), the second of the two hypotheses focussed on the relationship between costs and functional outcomes over time, with the prediction that overall costs and OOP expenditures will fall as the health benefits of treatment take hold and the need for specialized, general or indigenous care and support diminishes. A trend for greater cost reductions among those whose functional capacity had improved was seen across all sites, and differences reached statistical significance in Nepal. In two of the sites—Ethiopia and Uganda—a significant reduction in service costs and OOP expenditures was observed over time, and this held for different disorders and categories of service use, partly because of lower negotiated fees for essential psychotropic medications. Elsewhere, the trend was fluctuating or increasing. Analysis of the depression cohort in South Africa, e.g. clearly points to the specific investment made in improving access to mental health care and treatment in the 3 months between baseline and mid-line assessment, including an 8-week counselling intervention requiring service users to make additional clinic visits; at end-line assessment, costs fell back again towards baseline levels. In Nepal, analysis by disorder showed a large reduction in costs for the psychosis and epilepsy cohorts by mid-line assessment (undertaken 6 months after baseline for these conditions); by contrast, the depression and alcohol use disorder cohorts showed no appreciable change by mid-line assessment (undertaken 3 months after baseline for these conditions) and an increased cost associated with general health care services by end-line assessment, such as follow-up visits in PHC. A similar pattern was seen for these cohorts in the Indian site.

Use of an observational study design for assessing and understanding these relationships between resource costs and outcomes over time has a range of advantages and limitations. On the positive side, it provides for a naturalistic follow-up of how individuals with a range of mental health conditions responded to the increased supply of and access to local mental health care across diverse settings; however, it did not allow us to compare the specific effect of intervention package components for the different mental disorders and as a result, the primary interest was in testing associations rather than a causal relationship between resource costs and functional outcomes. Furthermore, the unit of analysis and data collection in this study was a cohort of individuals identified as having a mental disorder, rather than the household or population level, which restricted our capacity to link health service costs per cohort member to overall implementation costs in the study sites and to link OOP expenditures to estimates of catastrophic health spending at the household level. Such analyses require additional data points and are the subject of separate studies undertaken as part of or alongside PRIME (Lund *et al.*, 2019).

Another feature of the PRIME study was that the composition and implementation of district mental health care plans and cohort study protocols differed according to locally defined needs, priorities and capacities (Hanlon *et al.*, 2016; Baron *et al.*, 2018). Such a flexible approach is a key to meeting the challenge of mental health service development at a local level but means that packages of care and their costs or outcomes could not be compared on a like-with-like basis. Furthermore, the socio-economic environment itself differed appreciably between participating sites, including the relative affluence of the local population, the level of mental and general health care development, the degree of service accessibility and the extent of financial protection afforded to affected households. The mixed or partial support for our key hypotheses needs to be understood in the light of this heterogeneous set of service environments.

Alongside further observational or modelling studies of the inputs, process and outputs associated with mental health service scale-up, there is a consequent need for more trial-based cost-effectiveness studies in specific country settings that can more precisely identify the relative efficiency of different intervention strategies in improving health, social and economic outcomes for persons with priority MNS disorders.

## Conclusion

Analysis of service costs, private expenditures and their relationship with functional outcome within a multinational observational study provides the opportunity to gain new insights into the way resources are allocated, used and paid for in efforts to scale-up mental health care in a diverse range of settings; this study has generated indicative but not strong evidence across five participating sites for a reduction in overall service costs and OOP spending, especially for persons with improved levels of functioning.

## Supplementary Material

czz182_Supplementary_Data
