# Supplementary material for: Health service costs and their association with functional impairment among adults receiving integrated mental health care in five low- and middle-income countries: the PRIME cohort study
Source: Health Policy Plan. 2020 Mar 9;35(5):567–76. doi: 10.1093/heapol/czz182 (PMC11318687; doi:10.1093/heapol/czz182)
Supplement: czz182_Supplementary_Data [file heapol_35_5_567_s1.zip › czz182-Suppl_Data/PRIME Cohort study of service costs (HPP webtables).docx]

***Web Table 1. Demographic characteristics of the cohort samples recruited in each country***

| ETHIOPIA | DEP (n=0) | |  | AUD (n=30) | |  | PSY (n=266) | |  | EPI (n=244) | |
| --- | --- | --- | --- | --- | --- | --- | --- | --- | --- | --- | --- |
|  | N | % |  | N | % |  | N | % |  | N | % |
| Sex |  |  |  |  |  |  |  |  |  |  |  |
| Female | - | - |  | 1 | 3.3 |  | 114 | 42.9 |  | 98 | 40.2 |
| Age |  |  |  |  |  |  |  |  |  |  |  |
| 16-25 years | - | - |  | 3 | 10.0 |  | 76 | 28.6 |  | 96 | 39.3 |
| 26-35 years | - | - |  | 9 | 30.0 |  | 75 | 28.2 |  | 64 | 26.2 |
| 36-50 years | - | - |  | 10 | 33.3 |  | 82 | 30.8 |  | 63 | 25.8 |
| 51 years or more | - | - |  | 8 | 26.7 |  | 33 | 12.4 |  | 21 | 8.6 |
| Marital status |  |  |  |  |  |  |  |  |  |  |  |
| Has a partner | - | - |  | 26 | 86.7 |  | 100 | 37.6 |  | 107 | 43.9 |
| Educational level |  |  |  |  |  |  |  |  |  |  |  |
| Uneducated/illiterate | - | - |  | 18 | 60.0 |  | 139 | 52.4 |  | 146 | 60.1 |
| Non-formal/less than primary | - | - |  | 6 | 20.0 |  | 46 | 17.4 |  | 49 | 20.2 |
| Primary school and above | - | - |  | 6 | 20.0 |  | 80 | 30.2 |  | 48 | 19.7 |
| Employment |  |  |  |  |  |  |  |  |  |  |  |
| Employed | - | - |  | 27 | 93.1 |  | - | - |  | - | - |
| Food insecure | - | - |  | 1 | 3.3 |  | - | - |  | - | - |

| INDIA | DEP (n=258) | |  | AUD (n=205) | |  | PSY (n=39) | |  | EPI (n=0) | |
| --- | --- | --- | --- | --- | --- | --- | --- | --- | --- | --- | --- |
|  | N | % |  | N | % |  | N | % |  | N | % |
| Sex |  |  |  |  |  |  |  |  |  |  |  |
| Female | 159 | 61.6 |  | 1 | 0.5 |  | 7 | 36.8 |  | - | - |
| Age |  |  |  |  |  |  |  |  |  |  |  |
| 16-25 years | 59 | 22.9 |  | 24 | 11.7 |  | 5 | 26.3 |  | - | - |
| 26-35 years | 68 | 26.4 |  | 60 | 29.3 |  | 6 | 31.6 |  | - | - |
| 36-50 years | 97 | 37.6 |  | 82 | 40.0 |  | 6 | 31.6 |  | - | - |
| 51 years or more | 34 | 13.2 |  | 39 | 19.0 |  | 2 | 10.5 |  | - | - |
| Marital status |  |  |  |  |  |  |  |  |  |  |  |
| Has a partner | 230 | 89.2 |  | 197 | 96.1 |  | 13 | 68.4 |  | - | - |
| Educational level |  |  |  |  |  |  |  |  |  |  |  |
| Uneducated/illiterate | 87 | 33.7 |  | 48 | 23.4 |  | 5 | 26.3 |  | - | - |
| Non-formal/less than primary | 56 | 21.7 |  | 57 | 27.8 |  | 2 | 10.5 |  | - | - |
| Primary school and above | 115 | 44.6 |  | 100 | 48.8 |  | 12 | 63.2 |  | - | - |
| Employment |  |  |  |  |  |  |  |  |  |  |  |
| Employed | 128 | 49.6 |  | 193 | 94.2 |  | 10 | 52.6 |  | - | - |
| Food insecure | 10 | 3.9 |  | 11 | 5.4 |  | 1 | 5.3 |  | - | - |

| NEPAL | DEP (n=129) | |  | AUD (n=170) | |  | PSY (n=93) | |  | EPI (n=41) | |
| --- | --- | --- | --- | --- | --- | --- | --- | --- | --- | --- | --- |
|  | N | % |  | N | % |  | N | % |  | N | % |
| Sex |  |  |  |  |  |  |  |  |  |  |  |
| Female | 110 | 85.3 |  | 26 | 15.3 |  | 44 | 47.3 |  | 16 | 39.0 |
| Age |  |  |  |  |  |  |  |  |  |  |  |
| 16-25 years | 17 | 13.2 |  | 4 | 2.4 |  | 13 | 14.0 |  | 6 | 14.6 |
| 26-35 years | 35 | 29.1 |  | 46 | 27.1 |  | 20 | 21.5 |  | 15 | 36.6 |
| 36-50 years | 42 | 32.6 |  | 77 | 45.3 |  | 39 | 41.9 |  | 14 | 34.2 |
| 51 years or more | 35 | 27.1 |  | 43 | 25.3 |  | 21 | 22.6 |  | 6 | 14.6 |
| Marital status |  |  |  |  |  |  |  |  |  |  |  |
| Has a partner | 105 | 81.4 |  | 161 | 94.7 |  | 63 | 67.7 |  | 26 | 63.4 |
| Educational level |  |  |  |  |  |  |  |  |  |  |  |
| Uneducated/illiterate | 37 | 28.7 |  | 36 | 21.2 |  | 22 | 23.7 |  | 10 | 24.4 |
| Non-formal/less than primary | 36 | 27.9 |  | 32 | 18.8 |  | 37 | 39.8 |  | 5 | 12.2 |
| Primary school and above | 56 | 43.4 |  | 102 | 60.0 |  | 34 | 36.6 |  | 26 | 63.4 |
| Employment |  |  |  |  |  |  |  |  |  |  |  |
| Employed | 37 | 28.7 |  | 134 | 78.8 |  | 29 | 31.2 |  | 16 | 39.0 |
| Food insecure | 122 | 94.6 |  | 165 | 97.1 |  | 3 | 3.2 |  | 41 | 100.0 |

|  | DEP (n=216) | |  | AUD (n=0) | |  | PSY (n=29) | |  | EPI (n=0) | |
| --- | --- | --- | --- | --- | --- | --- | --- | --- | --- | --- | --- |
| SOUTH AFRICA | N | % |  | N | % |  | N | % |  | N | % |
| Sex |  |  |  |  |  |  |  |  |  |  |  |
| Female | 174 | 80.6 |  | - | - |  | 10 | 34.5 |  | - | - |
| Age |  |  |  |  |  |  |  |  |  |  |  |
| 16-25 years | 25 | 11.6 |  | - | - |  | 3 | 10.3 |  | - | - |
| 26-35 years | 50 | 23.2 |  | - | - |  | 8 | 27.6 |  |  |  |
| 36-50 years | 63 | 29.2 |  | - | - |  | 7 | 24.1 |  |  |  |
| 51 years or more | 78 | 36.1 |  | - | - |  | 11 | 37.9 |  |  |  |
| Marital status |  |  |  |  |  |  |  |  |  |  |  |
| Has a partner | 105 | 48.6 |  | - | - |  | 6 | 20.7 |  | - | - |
| Educational level |  |  |  |  |  |  |  |  |  |  |  |
| Uneducated/illiterate | 8 | 3.7 |  | - | - |  | 1 | 3.5 |  | - | - |
| Non-formal/less than primary | 54 | 25.0 |  | - | - |  | 15 | 51.7 |  | - | - |
| Primary school and above | 154 | 71.3 |  | - | - |  | 13 | 44.8 |  | - | - |
| Employment |  |  |  |  |  |  |  |  |  |  |  |
| Employed | 59 | 27.3 |  | - | - |  | 1 | 3.5 |  | - | - |
| Food insecure | 95 | 45.0 |  | - | - |  | 13 | 44.8 |  | - | - |

| UGANDA | DEP (n=63) | |  | AUD (n=0) | |  | PSY (n=51) | |  | EPI (n=181) | |
| --- | --- | --- | --- | --- | --- | --- | --- | --- | --- | --- | --- |
|  | N | % |  | N | % |  | N | % |  | N | % |
| Sex |  |  |  |  |  |  |  |  |  |  |  |
| Female | 47 | 74.6 |  | - | - |  | 23 | 45.1 |  | 93 | 51.4 |
| Age |  |  |  |  |  |  |  |  |  |  |  |
| 16-25 years | 9 | 14.3 |  | - | - |  | 19 | 37.3 |  | 96 | 53.0 |
| 26-35 years | 14 | 22.2 |  | - | - |  | 17 | 33.3 |  | 57 | 31.5 |
| 36-50 years | 28 | 44.4 |  | - | - |  | 13 | 25.5 |  | 24 | 13.3 |
| 51 years or more | 12 | 19.1 |  | - | - |  | 2 | 3.9 |  | 4 | 2.2 |
| Marital status |  |  |  |  |  |  |  |  |  |  |  |
| Has a partner | 39 | 61.9 |  | - | - |  | 15 | 29.4 |  | 28 | 15.5 |
| Educational level |  |  |  |  |  |  |  |  |  |  |  |
| Uneducated/illiterate | 15 | 23.8 |  | - | - |  | 1 | 2.0 |  | 44 | 24.3 |
| Non-formal/less than primary | 35 | 55.6 |  | - | - |  | 21 | 41.2 |  | 116 | 64.1 |
| Primary school and above | 13 | 20.6 |  | - | - |  | 29 | 56.9 |  | 21 | 11.6 |
| Employment |  |  |  |  |  |  |  |  |  |  |  |
| Employed | 29 | 46.0 |  | - | - |  | 17 | 33.3 |  | 53 | 29.3 |
| Food insecure | 15 | 23.8 |  | - | - |  | 11 | 21.6 |  | 36 | 19.9 |

***Web Table 2. Health care, travel and time costs (per 3 months) at baseline assessment, per country and cohort (US$, 2015)***

| **ETHIOPIA** | | **AUD** | | | **Psychosis** | | | **Epilepsy** | | |
| --- | --- | --- | --- | --- | --- | --- | --- | --- | --- | --- |
|  | | N | Mean | SD | N | Mean | SD | N | Mean | SD |
| Service cost, by type of care/service | | 30 | 8.34 | 38.48 | 266 | 7.30 | 24.64 | 244 | 3.67 | 11.80 |
| - Inpatient care | | 30 | 0.00 | 0.00 | 266 | 2.55 | 14.18 | 244 | 0.47 | 3.42 |
| - Outpatient care | | 30 | 8.34 | 38.48 | 266 | 4.76 | 18.15 | 244 | 3.20 | 11.31 |
| - - Mental health services | | 30 | 0.00 | 0.00 | 266 | 0.66 | 2.33 | 244 | 0.82 | 3.61 |
| - - General health services | | 30 | 7.93 | 39.01 | 266 | 1.50 | 37.01 | 244 | 1.71 | 9.73 |
| - - Indigenous / traditional services | | 30 | 0.41 | 1.65 | 266 | 2.59 | 18.02 | 244 | 0.68 | 4.30 |
| - Medication | | - | - | - | - | - | - | - | - | - |
|  | |  |  |  |  |  |  |  |  |  |
| Travel time and costs | |  |  |  |  |  |  |  |  |  |
| - Accessing / waiting for services | | 30 | 0.29 | 0.57 | 266 | 2.54 | 5.98 | 244 | 2.22 | 4.52 |
| - Travel payments | | 30 | 4.21 | 10.29 | 266 | 13.55 | 46.91 | 244 | 9.41 | 60.35 |
|  | |  |  |  |  |  |  |  |  |  |
| Total OOP expenditure by households | | 30 | 12.50 | 47.75 | 266 | 19.57 | 61.42 | 244 | 12.04 | 63.37 |
|  | | | | | | | | | | |
| Service cost, by level of functional impairment | | | | | | | | | | |
| - Lowest (<85^th^ percentile) | | 25 | 9.83 | 42.13 | 48 | 3.72 | 8.86 | 97 | 2.57 | 2.91 |
| - Higher (>=85^th^ percentile) | | 5 | 0.90 | 1.70 | 215 | 8.16 | 27.03 | 147 | 4.40 | 14.40 |
| *Difference (95% CI)* | *Higher impairment* | *-8.93 (-24.66 to 6.80)* | | | ***4.44 (0.11 to 8.78) **** | | | *1.83 (-0.77 to 4.43)* | | |
|  | |  |  |  |  |  |  |  |  |  |
| Out-of-pocket expenditure, by level of functional impairment | | | | | | | | | | |
| - Lowest (<85^th^ percentile) | | 25 | 14.55 | 52.22 | 48 | 10.10 | 25.07 | 97 | 16.20 | 97.23 |
| - Higher (>=85^th^ percentile) | | 5 | 2.25 | 3.46 | 215 | 21.80 | 67.14 | 147 | 9.29 | 21.16 |
| *Difference (95% CI)* | *Higher impairment* | *-12.30 (-33.60 to 8.99)* | | | ***11.71 (0.26 to 23.15) **** | | | *-6.92 (-27.07 to 13.23)* | | |

^m^ marginal; * p<0.05; ** p<0.01;

| **INDIA** | | **Depression** | | | **AUD** | | | **Psychosis** | | |
| --- | --- | --- | --- | --- | --- | --- | --- | --- | --- | --- |
|  | | N | Mean | SD | N | Mean | SD | N | Mean | SD |
| Service cost, by type of care/service | | 258 | 2.99 | 7.61 | 205 | 2.50 | 10.46 | 39 | 5.65 | 9.70 |
| - Inpatient care | | 258 | 1.14 | 7.40 | 205 | 1.21 | 10.17 | 39 | 2.06 | 9.56 |
| - Outpatient care | | 258 | 1.46 | 1.65 | 205 | 0.92 | 1.06 | 39 | 2.90 | 2.97 |
| - - Mental health services | | 258 | 0.26 | 0.80 | 205 | 0.21 | 0.50 | 39 | 1.42 | 2.75 |
| - - General health services | | 258 | 1.01 | 1.32 | 205 | 0.62 | 0.77 | 39 | 1.04 | 1.18 |
| - - Indigenous / traditional services | | 258 | 0.18 | 0.58 | 205 | 0.10 | 0.36 | 39 | 0.43 | 1.38 |
| - Medication | | 258 | 0.39 | 0.51 | 205 | 0.37 | 0.50 | 39 | 0.68 | 1.05 |
|  | |  |  |  |  |  |  |  |  |  |
| Travel time and costs | |  |  |  |  |  |  |  |  |  |
| - Accessing / waiting for services | | 258 | 1.00 | 1.22 | 205 | 0.88 | 1.42 | 39 | 1.37 | 1.51 |
| - Travel payments | | 258 | 0.62 | 1.19 | 205 | 0.47 | 0.67 | 39 | 1.06 | 1.39 |
|  | |  |  |  |  |  |  |  |  |  |
| Total OOP expenditure by households | | 258 | 2.23 | 6.27 | 205 | 1.86 | 7.23 | 39 | 4.60 | 9.16 |
|  | |  |  |  |  |  |  |  |  |  |
| Service cost, by level of functional impairment (85^th^ percentile) | | | | | | | | | | |
| - Lowest (<85^th^ percentile) | | 151 | 1.84 | 3.45 | 151 | 1.51 | 3.31 | 7 | 2.50 | 2.06 |
| - Higher (>=85^th^ percentile) | | 107 | 4.62 | 10.90 | 54 | 5.28 | 19.47 | 12 | 3.81 | 3.66 |
| *Difference (95% CI)* | *Higher impairment* | ***2.78 (0.58 to 4.98) **** | | | *3.77 (-1.22 to 8.77)* | | | *1.31 (-1.16 to 3.78)* | | |
|  | | | | | | | | | | |
| Out-of-pocket expenditure, by level of impairment (85^th^ percentile) | | | | | | | | | | |
| - Lowest (<85^th^ percentile) | | 151 | 1.43 | 3.55 | 151 | 1.18 | 1.96 | 7 | 2.42 | 2.69 |
| - Higher (>=85^th^ percentile) | | 107 | 3.34 | 8.69 | 54 | 3.76 | 13.62 | 12 | 2.46 | 2.89 |
| *Difference (95% CI)* | *Higher impairment* | ***1.91 (0.14 top 3.68) **** | | | *2.58 (-1.06 to 6.22)* | | | *0.04 (-2.49 to 2.56)* | | |

^m^ marginal; * p<0.05; ** p<0.01; *** p<0.001

| **NEPAL** | | **Depression** | | | **AUD** | | | **Psychosis** | | | **Epilepsy** | | |
| --- | --- | --- | --- | --- | --- | --- | --- | --- | --- | --- | --- | --- | --- |
|  | | N | Mean | SD | N | Mean | SD | N | Mean | SD | N | Mean | SD |
| Service cost, by type of care/service | | 129 | 7.47 | 19.15 | 170 | 5.06 | 17.07 | 93 | 19.52 | 40.61 | 41 | 18.67 | 55.49 |
| - Inpatient care | | 129 | 3.33 | 15.17 | 170 | 2.33 | 16.43 | 93 | 6.86 | 31.49 | 41 | 5.37 | 27.74 |
| - Outpatient care | | 129 | 3.41 | 11.36 | 170 | 1.76 | 5.10 | 93 | 8.29 | 24.05 | 41 | 10.61 | 49.11 |
| - - Mental health services | | 129 | 0.03 | 0.29 | 170 | 0.24 | 3.05 | 93 | 3.21 | 6.72 | 41 | 8.59 | 49.27 |
| - - General health services | | 129 | 2.26 | 9.19 | 170 | 1.08 | 3.13 | 93 | 1.60 | 4.19 | 41 | 1.86 | 4.21 |
| - - Indigenous / traditional services | | 129 | 1.12 | 5.73 | 170 | 0.44 | 2.83 | 93 | 3.48 | 22.70 | 41 | 0.16 | 1.06 |
| - Medication | | 129 | 0.73 | 0.74 | 170 | 0.97 | 0.71 | 93 | 4.36 | 3.94 | 41 | 2.69 | 2.93 |
|  | |  |  |  |  |  |  |  |  |  |  |  |  |
| Travel time and costs | |  |  |  |  |  |  |  |  |  |  |  |  |
| - Accessing / waiting for services | | 129 | 1.67 | 3.02 | 170 | 0.92 | 2.31 | 93 | 3.44 | 6.81 | 41 | 2.53 | 6.14 |
| - Travel payments | | 129 | 0.45 | 1.63 | 170 | 0.18 | 0.82 | 93 | 1.64 | 4.23 | 41 | 0.93 | 4.23 |
|  | |  |  |  |  |  |  |  |  |  |  |  |  |
| Total OOP expenditure by households | | 129 | 7.66 | 18.97 | 170 | 5.03 | 16.70 | 93 | 20.46 | 41.94 | 41 | 18.74 | 57.24 |
|  | |  |  |  |  |  |  |  |  |  |  |  |  |
| **Service cost, by level of functional impairment (85^th^ percentile)** | | | | | | | | | | | | | |
| - Lowest (<85^th^ percentile) | | 41 | 7.71 | 21.84 | 118 | 3.99 | 11.56 | 45 | 14.63 | 26.34 | 29 | 23.11 | 65.44 |
| - Higher (>=85^th^ percentile) | | 88 | 7.36 | 17.89 | 52 | 7.47 | 25.50 | 48 | 24.09 | 50.35 | 12 | 7.95 | 10.91 |
| *Difference (95% CI)* | *Higher impairment* | *-0.35 (-8.12 to 7.42)* | | | *3.48 (-3.46 to 10.42)* | | | *9.47 (-7.30 to 26.21)* | | | *-15.16 (-39.29 to 8.96)* | | |
|  | | | | | | | | | | | | | |
| **Out-of-pocket expenditure, by level of impairment (85^th^ percentile)** | | | | | | | | | | | | | |
| - Lowest (<85^th^ percentile) | | 41 | 7.68 | 21.65 | 118 | 3.88 | 10.71 | 45 | 14.69 | 25.97 | 29 | 23.14 | 67.55 |
| - Higher (>=85^th^ percentile) | | 88 | 7.65 | 17.72 | 52 | 7.63 | 25.51 | 48 | 25.88 | 52.44 | 12 | 8.10 | 11.27 |
| *Difference (95% CI)* | *Higher impairment* | *-0.02 (-7.41 to 7.36)* | | | *3.75 (-3.54 to 11.04)* | | | *11.18 (-5.46 to 27.83)* | | | *-15.03 (-41.51 to 11.44)* | | |

^m^ marginal; * p<0.05; ** p<0.01; *** p<0.001

| SOUTH AFRICA | | **Depression** | | | **Psychosis** | | |
| --- | --- | --- | --- | --- | --- | --- | --- |
|  | | N | Mean | SD | N | Mean | SD |
| Service cost, by type of care/service | | 216 | 19.30 | 29.86 | 29 | 50.45 | 84.03 |
| - Inpatient care | | 216 | 5.58 | 25.29 | 29 | 23.23 | 64.12 |
| - Outpatient care | | 216 | 13.32 | 16.00 | 29 | 13.74 | 34.74 |
| - - Mental health services | | 216 | 1.59 | 7.25 | 29 | 5.22 | 22.19 |
| - - General health services | | 216 | 10.60 | 12.06 | 29 | 7.90 | 14.32 |
| - - Indigenous / traditional services | | 216 | 1.43 | 6.37 | 29 | 0.61 | 3.04 |
| - Medication | | 216 | 0.40 | 2.53 | 29 | 13.68 | 16.57 |
|  | |  |  |  |  |  |  |
| Travel time and costs | |  |  |  |  |  |  |
| - Accessing / waiting for services | | 216 | 12.11 | 13.80 | 29 | 16.55 | 21.33 |
| - Travel payments | | 216 | 1.21 | 4.38 | 29 | 0.95 | 2.97 |
|  | |  |  |  |  |  |  |
| Total OOP expenditure by households | | 216 | 1.21 | 4.38 | 29 | 0.95 | 2.98 |
|  | |  |  |  |  |  |  |
| **Service cost, by level of functional impairment (85^th^ percentile)** | | | | | | | |
| - Lowest (<85^th^ percentile) | | 90 | 16.58 | 28.58 | 18 | 42.49 | 75.63 |
| - Higher (>=85^th^ percentile) | | 156 | 21.24 | 30.70 | 11 | 64.01 | 98.66 |
| *Difference (95% CI)* | *Higher impairment* | *4.66 (-3.38 to 12.70)* | | | *21.52 (-41.92 to 84.96)* | | |
|  | |  |  |  |  |  |  |
| **Out-of-pocket expenditure, by level of impairment (85^th^ percentile)** | | | | | | | |
| - Lowest (<85^th^ percentile) | | 90 | 5.66 | 11.24 | 18 | 13.14 | 14.33 |
| - Higher (>=85^th^ percentile) | | 126 | 5.42 | 12.81 | 11 | 18.87 | 20.15 |
| *Difference (95% CI)* | *Higher impairment* | *-0.23 (-3.54 to 3.07)* | | | *5.73 (-8.38 to 19.84)* | | |

^m^ marginal; * p<0.05; ** p<0.01; *** p<0.001

| UGANDA | | **Depression** | | | **Psychosis** | | | **Epilepsy** | | |
| --- | --- | --- | --- | --- | --- | --- | --- | --- | --- | --- |
|  | | N | Mean | SD | N | Mean | SD | N | Mean | SD |
| Service cost, by type of care/service | | 63 | 6.69 | 8.51 | 51 | 12.30 | 19.49 | 181 | 5.12 | 7.13 |
| - Inpatient care | | 63 | 1.81 | 5.58 | 51 | 4.82 | 13.35 | 181 | 0.95 | 4.43 |
| - Outpatient care | | 63 | 3.47 | 6.26 | 51 | 6.07 | 14.06 | 181 | 2.39 | 5.36 |
| - - Mental health services | | 63 | 0.23 | 1.10 | 51 | 2.83 | 3.39 | 181 | 0.78 | 2.19 |
| - - General health services | | 63 | 2.94 | 5.87 | 51 | 0.57 | 1.70 | 181 | 0.36 | 0.90 |
| - - Indigenous / traditional services | | 63 | 0.30 | 1.91 | 51 | 2.67 | 13.62 | 181 | 1.24 | 4.83 |
| - Medication | | 63 | 1.42 | 1.73 | 51 | 1.41 | 1.52 | 181 | 1.78 | 2.35 |
|  | |  |  |  |  |  |  |  |  |  |
| Travel time and costs | |  |  |  |  |  |  |  |  |  |
| - Accessing / waiting for services | | 63 | 0.94 | 1.45 | 51 | 1.80 | 2.67 | 181 | 0.77 | 1.21 |
| - Travel payments | | 63 | 1.59 | 1.90 | 51 | 3.73 | 7.22 | 181 | 1.23 | 4.05 |
|  | |  |  |  |  |  |  |  |  |  |
| Total OOP expenditure by households | | 63 | 5.53 | 7.64 | 51 | 9.00 | 17.68 | 181 | 4.71 | 7.60 |
|  | |  |  |  |  |  |  |  |  |  |
| **Service cost, by level of functional impairment (85^th^ percentile)** | | | | | | | | | | |
| - Lowest (<85^th^ percentile) | | 10 | 7.93 | 8.31 | 20 | 6.60 | 6.75 | 95 | 4.55 | 5.13 |
| - Higher (>=85^th^ percentile) | | 53 | 6.46 | 8.60 | 31 | 15.97 | 23.85 | 86 | 5.74 | 8.81 |
| *Difference (95% CI)* | *Higher impairment* | *-1.47 (-7.15 to 4.21)* | | | ***9.38 (0.32 to 18.44) **** | | | *1.19 (-0.85 to 3.23)* | | |
|  | | | | | | | | | | |
| **Out-of-pocket expenditure, by level of impairment (85^th^ percentile)** | | | | | | | | | | |
| - Lowest (<85^th^ percentile) | | 10 | 5.29 | 2.71 | 20 | 3.98 | 4.81 | 95 | 3.93 | 4.74 |
| - Higher (>=85^th^ percentile) | | 53 | 5.57 | 8.26 | 31 | 12.24 | 21.87 | 86 | 5.57 | 9.81 |
| *Difference (95% CI)* | *Higher impairment* | *0.28 (-2.59 to 3.15)* | | | ***8.26 (0.27 to 16.25) **** | | | *1.64 (-0.58 to 3.87)* | | |

^m^ marginal; * p<0.05; ** p<0.01; *** p<0.001

***Web Table 3a - Health care costs and out-of-pocket payments over time in Ethiopia, by disease cohort and service component (US$, 2015)***

| **Ethiopia - AUD** | **All care** | | | **Mental health** | | | **General** | | | **Indigenous** | | |
| --- | --- | --- | --- | --- | --- | --- | --- | --- | --- | --- | --- | --- |
|  | N | Mean | SD | N | Mean | SD | N | Mean | SD | N | Mean | SD |
| **Total service cost per 3 months** | | | | | | | | | | | | |
| - Baseline | 30 | 8.34 | 38.48 | 30 | 0.00 | 0.00 | 30 | 7.93 | 37.01 | 30 | 0.41 | 1.65 |
| - Midline | 30 | 3.05 | 9.60 | 30 | 0.04 | 0.22 | 30 | 2.64 | 9.63 | 30 | 0.37 | 1.17 |
| - Endline | 29 | 0.24 | 0.65 | 29 | 0.08 | 0.45 | 29 | 0.16 | 0.51 | 29 | 0.00 | 0.00 |
| *Test of difference (coefficient, 95%CI)* | | | | | | | | | | | | |
| - *Midline - Baseline* | *-5.29 (-13.75 to 3.16)* | | | *0.03 (-0.01 to 0.07)* | | | *-5.29 (-13.23 to 2.65)* | | | *-0.04 (-0.65 to 0.57)* | | |
| - *Endline - Baseline* | *-8.22 (-18.24 to 1.80)* | | | *0.05 (-0.05 to 0.15)* | | | *-7.88 (-17.51 to 1.74)* | | | ***-0.42 (-0.88 to 0.05) ^m^*** | | |
|  | | | | | | | | | | | | |
| **Change in total service cost (endline – baseline)** | | | | | | | | | | | | |
| - Functioning worsens | 16 | -13.16 | 52.32 | 16 | 0.15 | 0.60 | 16 | -12.54 | 50.29 | 16 | -0.77 | 2.23 |
| - Functioning improves | 13 | -2.51 | 5.41 | 13 | 0.00 | 0.00 | 13 | -2.51 | 5.40 | 13 | 0.00 | 0.00 |
| *Test of association (β, 95% CI)* | ***-0.36 (-0.76 to 0.03) ^m^*** | | | *-* | | | *-0.21 (-0.49 to 0.07)* | | | ***-*** | | |
|  |  |  |  |  |  |  |  |  |  |  |  |  |
| **Total private, out-of-pocket cost per 3 months** | | | |  |  |  |  |  |  |  |  |  |
| - Baseline | 30 | 12.50 | 47.75 | 30 | 0.00 | 0.00 | 30 | 11.05 | 42.58 | 30 | 1.45 | 5.82 |
| - Midline | 30 | 3.98 | 11.94 | 30 | 0.06 | 0.34 | 30 | 3.54 | 12.01 | 30 | 0.37 | 1.17 |
| - Endline | 29 | 1.03 | 2.81 | 29 | 0.33 | 1.79 | 29 | 0.62 | 2.26 | 29 | 0.08 | 0.43 |
| *Test of difference (coefficient, 95%CI)* | | | | | | | | | | | | |
| - *Midline - Baseline* | *-8.52 (-19.04 to 2.00)* | | | *0.05 (-0.02 to 0.13)* | | | *-7.51 (-16.88 to 1.86)* | | | *-1.08 (-2.68 to 0.53)* | | |
| - *Endline - Baseline* | ***-11.65 (-23.95 to 0.64) ^m^*** | | | *0.21 (-0.20 to 0.61)* | | | ***-10.60 (-21.78 to 0.57) ^m^*** | | | *-1.38 (-3.07 to 0.30)* | | |
|  | | | | | | | | | | | | |
| **Change in out-of-pocket cost (endline – baseline)** | | | | | | | | | | | | |
| - Functioning worsens | 16 | -16.89 | 62.59 | 16 | 0.60 | 2.42 | 16 | -14.93 | 55.38 | 16 | -2.57 | 7.94 |
| - Functioning improves | 13 | -5.76 | 12.45 | 13 | 0.00 | 0.00 | 13 | -5.76 | 12.45 | 13 | 0.00 | 0.00 |
| *Test of association (β, 95% CI)* | ***-1.43 (-2.98 to 0.12) ^m^*** | | | *-* | | | *-0.72 (-1.83 to 0.38)* | | | *-* | | |

^m^ marginal; * p<0.05

| **Ethiopia – Psychosis**  **(Available on request from authors)** | **All care** | | | **Mental health** | | | **General** | | | **Indigenous** | | |
| --- | --- | --- | --- | --- | --- | --- | --- | --- | --- | --- | --- | --- |
|  | N | Mean | SD | N | Mean | SD | N | Mean | SD | N | Mean | SD |
| **Total service cost per 3 months** | | | | | | | | | | | | |
| - Baseline | 266 | 7.30 | 24.64 | 266 | 2.09 | 9.07 | 266 | 2.62 | 12.84 | 266 | 2.59 | 18.02 |
| - Midline | 218 | 1.94 | 7.33 | 218 | 0.71 | 5.38 | 218 | 0.83 | 3.71 | 218 | 0.40 | 3.65 |
| - Endline | 213 | 1.85 | 8.07 | 213 | 0.88 | 7.74 | 213 | 0.79 | 2.27 | 213 | 0.19 | 0.73 |
| *Test of difference (coefficient, 95%CI)* | | | | | | | | | | | | |
| - *Midline - Baseline* | ***-5.66 (-8.20 to -3.11) ****** | | | ***-1.33 (-2.40 to -0.26) **** | | | ***-1.90 (-3.21 to -0.59) ***** | | | ***-2.41 (-4.28 to -0.55) **** | | |
| - *Endline - Baseline* | ***-4.91 (-7.16 to -2.67) ****** | | | *-1.01 (-2.31 to 0.29)* | | | ***-1.73 (-2.92 to -0.54) ***** | | | ***-2.17 (-3.56 to -0.78) ***** | | |
|  | | | | | | | | | | | | |
| **Change in total service cost (endline – baseline)** | | | | | | | | | | | | |
| - Functioning worsens | 70 | -3.75 | 25.90 | 70 | -0.43 | 17.38 | 70 | -1.23 | 8.48 | 70 | -2.09 | 17.38 |
| - Functioning improves | 140 | -4.41 | 19.52 | 140 | -0.97 | 6.52 | 140 | -1.66 | 15.41 | 140 | -1.78 | 7.45 |
| *Test of association (β, 95% CI)* | *-1.64 (-4.60 to 1.33)* | | | *-1.42 (-4.54 to 1.69)* | | | *-0.07 (-0.63 to 0.49)* | | | *-0.16 (-0.39 to 0.08)* | | |
|  |  |  |  |  |  |  |  |  |  |  |  |  |
| **Total private, out-of-pocket cost per 3 months** | | | |  |  |  |  |  |  |  |  |  |
| - Baseline | 266 | 19.57 | 61.42 | 266 | 3.52 | 11.90 | 266 | 6.75 | 27.97 | 266 | 9.29 | 46.75 |
| - Midline | 218 | 3.83 | 10.79 | 218 | 1.13 | 5.14 | 218 | 1.56 | 3.94 | 218 | 1.15 | 9.01 |
| - Endline | 213 | 3.88 | 7.19 | 213 | 0.98 | 5.39 | 213 | 2.06 | 4.49 | 213 | 0.83 | 3.19 |
| *Test of difference (coefficient, 95%CI)* | | | | | | | | | | | | |
| - *Midline - Baseline* | ***-16.51 (-22.46 to -10.56) ****** | | | ***-2.37 (-3.60 to -1.13) ****** | | | ***-5.47 (-8.14 to -2.79) ****** | | | ***-8.65 (-13.37 to -3.92) ****** | | |
| - *Endline - Baseline* | ***-15.80 (-21.50 to -10.09) ****** | | | ***-2.40 (-3.71 to -1.09) ****** | | | ***-4.77 (-7.42 to -2.13) ****** | | | ***-8.58 (-12.83 to -4.34) ****** | | |
|  | | | | | | | | | | | | |
| **Change in out-of-pocket cost (endline – baseline)** | | | | | | | | | | | | |
| - Functioning worsens | 70 | -17.09 | 72.48 | 70 | -3.03 | 18.74 | 70 | -5.32 | 26.65 | 70 | -8.75 | 55.26 |
| - Functioning improves | 140 | -15.58 | 57.11 | 140 | -1.89 | 7.87 | 140 | -4.64 | 32.53 | 140 | -9.03 | 40.09 |
| *Test of association (β, 95% CI)* | ***-2.82 (-5.42 to -0.21) **** | | | *-1.41 (-3.52 to 0.71)* | | | *-0.48 (-1.47 to 0.51)* | | | ***-0.98 (-2.13 to 0.17) ^m^*** | | |

^m^ marginal; * p<0.05

| **Ethiopia - epilepsy** | **All care** | | | **Mental health** | | | | **General** | | | | **Indigenous** | | |
| --- | --- | --- | --- | --- | --- | --- | --- | --- | --- | --- | --- | --- | --- | --- |
|  | N | Mean | SD | N | Mean | SD | N | | Mean | SD | N | | Mean | SD |
| **Total service cost per 3 months** | | | | | | | | | | | | | | |
| - Baseline | 244 | 3.67 | 11.80 | 244 | 0.99 | 4.16 | 244 | | 2.01 | 10.12 | 244 | | 0.68 | 4.30 |
| - Midline | 119 | 1.69 | 3.22 | 119 | 0.18 | 0.49 | 119 | | 1.43 | 3.21 | 119 | | 0.08 | 0.50 |
| - Endline | 204 | 1.64 | 6.14 | 204 | 0.24 | 1.98 | 204 | | 1.34 | 5.83 | 204 | | 0.07 | 0.60 |
| *Test of difference (coefficient, 95%CI)* | | | | | | | | | | | | | | |
| - *Midline - Baseline* | ***-1.70 (-2.85 to -0.55) ***** | | | ***-0.75 (-1.17 to -0.33) ****** | | | | *-0.30 (-1.22 to 0.61)* | | | | ***-0.65 (-1.11 to -0.19) ***** | | |
| - *Endline - Baseline* | ***-1.80 (-3.10 to -0.49) ***** | | | ***-0.63 (-1.08 to -0.17) ***** | | | | *-0.54 (-1.80 to 0.72)* | | | | ***-0.63 (-1.07 to -0.20) ***** | | |
|  | | | | | | | | | | | | | | |
| **Change in total service cost (endline – baseline)** | | | | | | | | | | | | | | |
| - Functioning worsens | 96 | -3.04 | 16.43 | 96 | -0.76 | 5.72 | 96 | | -1.81 | 15.41 | 96 | | -0.47 | 3.23 |
| - Functioning improves | 106 | -0.33 | 6.65 | 106 | -0.29 | 1.38 | 106 | | 0.82 | 7.53 | 106 | | -0.85 | 5.48 |
| *Test of association (β, 95% CI)* | *0.55 (-1.24 to 2.33)* | | | *-0.35 (-0.95 to 0.24)* | | | | *0.77 (-0.84 to 2.37)* | | | | ***-0.15 (-0.32 to 0.02) ^m^*** | | |
|  |  |  |  |  |  |  |  | |  |  |  | |  |  |
| **Total private, out-of-pocket cost per 3 months** | | | |  |  |  |  | |  |  |  | |  |  |
| - Baseline | 244 | 12.04 | 63.37 | 244 | 6.38 | 61.06 | 244 | | 3.54 | 10.67 | 244 | | 2.08 | 9.45 |
| - Midline | 119 | 4.48 | 8.34 | 119 | 1.34 | 5.22 | 119 | | 2.92 | 6.44 | 119 | | 0.23 | 1.53 |
| - Endline | 204 | 2.94 | 7.06 | 204 | 0.31 | 2.03 | 204 | | 2.36 | 6.57 | 204 | | 0.26 | 1.54 |
| *Test of difference (coefficient, 95%CI)* | | | | | | | | | | | | | | |
| - *Midline - Baseline* | ***-8.91 (-17.44 to -0.39) **** | | | *-6.57 (-14.85 to 1.74)* | | | | *-0.21 (-1.66 to 1.24)* | | | | ***-1.87 (-3.03 to -0.71) ***** | | |
| - *Endline - Baseline* | ***-8.84 (-15.25 to -2.42) ***** | | | ***-6.26 (-12.26 to -0.27) **** | | | | *-0.45 (-1.57 to 0.68)* | | | | ***-1.72 (-2.57 to -0.86) ****** | | |
|  | | | | | | | | | | | | | | |
| **Change in out-of-pocket cost (endline – baseline)** | | | | | | | | | | | | | | |
| - Functioning worsens | 96 | -14.38 | 98.27 | 96 | -12.53 | 97.00 | 96 | | -0.85 | 8.70 | 96 | | -0.98 | 5.20 |
| - Functioning improves | 106 | -3.00 | 14.91 | 106 | -1.39 | 4.98 | 106 | | 0.56 | 9.92 | 106 | | -2.18 | 11.38 |
| *Test of association (β, 95% CI)* | *0.67 (-1.30 to 2.65)* | | | *-0.16 (-0.76 to 0.44)* | | | | *0.98 (-0.82 to 2.77)* | | | | *-0.22 (-0.65 to 0.22)* | | |

^m^ marginal; * p<0.05

***Web Table 3b - Health care costs and out-of-pocket payments over time in India, by disease cohort and service component (US$, 2015)***

| **India - Depression** | **All care** | | | **Mental health** | | | **General** | | | **Indigenous** | | |
| --- | --- | --- | --- | --- | --- | --- | --- | --- | --- | --- | --- | --- |
|  | N | Mean | SD | N | Mean | SD | N | Mean | SD | N | Mean | SD |
| **Total service cost per 3 months** | | | | | | | | | | | | |
| - Baseline | 258 | 2.99 | 7.61 | 258 | 0.65 | 1.09 | 258 | 2.16 | 7.58 | 258 | 0.18 | 0.58 |
| - Midline | 239 | 2.83 | 5.43 | 239 | 0.44 | 1.09 | 239 | 2.05 | 5.20 | 239 | 0.33 | 1.06 |
| - Endline | 222 | 6.23 | 22.34 | 222 | 0.11 | 0.93 | 222 | 5.80 | 22.39 | 222 | 0.32 | 0.81 |
| *Test of difference (coefficient, 95%CI)* | | | | | | | | | | | | |
| - *Midline - Baseline* | *-0.12 (-1.06 to 0.82)* | | | ***-0.22 (-0.36 to -0.07) ***** | | | *-0.05 (-0.94 to 0.85)* | | | ***0.14 (0.01 to 0.28) **** | | |
| - *Endline - Baseline* | ***3.29 (0.41 to 6.18) **** | | | ***-0.55 (-0.71 to -0.40) ****** | | | ***3.72 (0.79 to 6.64) **** | | | ***0.13 (0.02 to 0.24) **** | | |
|  | | | | | | | | | | | | |
| **Change in total service cost (endline – baseline)** | | | | | | | | | | | | |
| - Functioning worsens | 103 | 4.19 | 27.97 | 103 | -0.50 | 1.60 | 103 | 4.52 | 27.91 | 103 | 0.17 | 0.89 |
| - Functioning improves | 119 | 2.63 | 17.55 | 119 | -0.61 | 1.19 | 119 | 3.16 | 17.47 | 119 | 0.08 | 0.98 |
| *Test of association (β, 95% CI)* | *-1.23 (-7.43 to 4.97)* | | | *-0.02 (-0.25 to 0.21)* | | | *-1.21 (-6.98 to 4.56)* | | | *0.02 (-0.19 to 0.23)* | | |
|  |  |  |  |  |  |  |  |  |  |  |  |  |
| **Total private, out-of-pocket cost per 3 months** | | | |  |  |  |  |  |  |  |  |  |
| - Baseline | 258 | 2.23 | 6.27 | 258 | 0.54 | 0.84 | 258 | 1.38 | 6.17 | 258 | 0.30 | 0.96 |
| - Midline | 239 | 1.77 | 3.09 | 239 | 0.31 | 0.90 | 239 | 1.08 | 2.72 | 239 | 0.39 | 1.23 |
| - Endline | 222 | 5.16 | 21.82 | 222 | 0.12 | 1.01 | 222 | 4.64 | 21.87 | 222 | 0.40 | 0.98 |
| *Test of difference (coefficient, 95%CI)* | | | | | | | | | | | | |
| - *Midline - Baseline* | *-0.39 (-1.04 to 0.25)* | | | ***-0.23 (-0.31 to -0.15) ****** | | | *-0.24 (-0.88 to 0.40)* | | | *0.08 (-0.09 to 0.26)* | | |
| - *Endline - Baseline* | ***2.96 (0.12 to 5.80) **** | | | ***-0.42 (-0.57 to -0.27) ****** | | | ***3.29 (0.38 to 6.20) **** | | | *0.09 (-0.05 to 0.24)* | | |
|  | | | | | | | | | | | | |
| **Change in out-of-pocket cost (endline – baseline)** | | | | | | | | | | | | |
| - Functioning worsens | 103 | 4.18 | 28.08 | 103 | -0.34 | 1.21 | 103 | 4.42 | 27.99 | 103 | 0.10 | 1.30 |
| - Functioning improves | 119 | 1.97 | 16.07 | 119 | -0.49 | 1.23 | 119 | 2.39 | 15.99 | 119 | 0.08 | 1.19 |
| *Test of association (β, 95% CI)* | *-1.77 (-7.57 to 4.02)* | | | *-0.02 (-0.25 to 0.22)* | | | *-1.87 (-7.67 to 3.92)* | | | *0.09 (-0.17 to 0.34)* | | |

^m^ marginal; * p<0.05; ** p<0.01; *** p<0.001

| **India - AUD** | **All care** | | | **Mental health** | | | **General** | | | **Indigenous** | | |
| --- | --- | --- | --- | --- | --- | --- | --- | --- | --- | --- | --- | --- |
|  | N | Mean | SD | N | Mean | SD | N | Mean | SD | N | Mean | SD |
| **Total service cost per 3 months** | | | | | | | | | | | | |
| - Baseline | 205 | 2.50 | 10.46 | 205 | 0.58 | 0.77 | 205 | 1.83 | 10.28 | 205 | 0.10 | 0.36 |
| - Midline | 191 | 3.09 | 14.97 | 191 | 0.39 | 0.93 | 191 | 2.35 | 14.92 | 191 | 0.35 | 1.31 |
| - Endline | 175 | 2.84 | 12.56 | 175 | 0.02 | 0.09 | 175 | 2.49 | 12.54 | 175 | 0.33 | 0.85 |
| *Test of difference (coefficient, 95%CI)* | | | | | | | | | | | | |
| - *Midline - Baseline* | *0.52 (-0.69 to 1.74)* | | | ***-0.20 (-0.34 to -0.05) ***** | | | *0.47 (-0.73 to 1.67)* | | | ***0.26 (0.07 to 0.44) ***** | | |
| - *Endline - Baseline* | *0.19 (-2.01 to 2.39)* | | | ***-0.57 (-0.65 to -0.48) ****** | | | *0.52 (-1.73 to 2.77)* | | | ***0.24 (0.10 to 0.37) ****** | | |
|  | | | | | | | | | | | | |
| **Change in total service cost (endline – baseline)** | | | | | | | | | | | | |
| - Functioning worsens | 100 | 1.27 | 13.51 | 100 | -0.45 | 0.60 | 100 | 1.46 | 13.49 | 100 | 0.26 | 0.80 |
| - Functioning improves | 75 | -1.36 | 20.62 | 75 | -0.74 | 1.03 | 75 | -0.84 | 20.44 | 75 | 0.21 | 1.03 |
| *Test of association (β, 95% CI)* | *0.10 (-3.59 to 3.80)* | | | *-0.02 (-0.04 to 0.01)* | | | *0.06 (-3.56 to 3.68)* | | | *0.05 (-0.23 to 0.33)* | | |
|  |  |  |  |  |  |  |  |  |  |  |  |  |
| **Total private, out-of-pocket cost per 3 months** | | | |  |  |  |  |  |  |  |  |  |
| - Baseline | 205 | 1.86 | 7.23 | 205 | 0.54 | 0.60 | 205 | 1.17 | 6.91 | 205 | 0.15 | 0.59 |
| - Midline | 191 | 2.74 | 15.04 | 191 | 0.25 | 0.47 | 191 | 2.09 | 14.98 | 191 | 0.41 | 1.55 |
| - Endline | 175 | 2.67 | 12.35 | 175 | 0.02 | 0.09 | 175 | 2.18 | 12.09 | 175 | 0.48 | 1.47 |
| *Test of difference (coefficient, 95%CI)* | | | | | | | | | | | | |
| - *Midline - Baseline* | *0.81 (-0.42 to 2.04)* | | | ***-0.30 (-0.38 to -0.22) ****** | | | *0.86 (-0.40 to 2.12)* | | | ***0.26 (0.03 to 0.48) **** | | |
| - *Endline - Baseline* | *0.70 (-1.49 to 2.89)* | | | ***-0.53 (-0.59 to -0.46) ****** | | | *0.87 (-1.17 to 2.92)* | | | ***0.34 (0.12 to 0.56) ***** | | |
|  | | | | | | | | | | | | |
| **Change in out-of-pocket cost (endline – baseline)** | | | | | | | | | | | | |
| - Functioning worsens | 100 | 1.42 | 12.78 | 100 | -0.45 | 0.59 | 100 | 1.41 | 12.39 | 100 | 0.45 | 1.71 |
| - Functioning improves | 75 | -0.27 | 16.79 | 75 | -0.63 | 0.65 | 75 | 0.14 | 16.52 | 75 | 0.22 | 1.33 |
| *Test of association (β, 95% CI)* | *0.41 (-3.44 to 4.25)* | | | *-0.02 (-0.04 to 0.01)* | | | *0.52 (-3.05 to 4.10)* | | | *-0.07 (-0.50 to 0.35)* | | |

^m^ marginal; * p<0.05; ** p<0.01; *** p<0.001

| **India - Psychosis** | **All care** | | | **Mental health** | | | **General** | | | **Indigenous** | | |
| --- | --- | --- | --- | --- | --- | --- | --- | --- | --- | --- | --- | --- |
|  | N | Mean | SD | N | Mean | SD | N | Mean | SD | N | Mean | SD |
| **Total service cost per 3 months** | | | | | | | | | | | | |
| - Baseline | 39 | 5.65 | 9.70 | 39 | 3.56 | 9.07 | 39 | 1.65 | 4.10 | 39 | 0.43 | 1.38 |
| - Midline | 35 | 7.99 | 23.87 | 35 | 6.33 | 23.86 | 35 | 1.43 | 4.00 | 35 | 0.23 | 0.85 |
| - Endline | 36 | 10.07 | 25.86 | 36 | 9.08 | 25.98 | 36 | 0.88 | 1.47 | 36 | 0.11 | 0.33 |
| *Test of difference (coefficient, 95%CI)* | | | | | | | | | | | | |
| - *Midline - Baseline* | *2.19 (-5.96 to 10.34)* | | | *2.65 (-5.79 to 11.10)* | | | *-0.27 (-1.89 to 1.34)* | | | *-0.22 (-0.68 to 0.24)* | | |
| - *Endline - Baseline* | *4.39 (-4.13 to 12.91)* | | | *5.51 (-3.31 to 14.33)* | | | *-0.78 (-1.89 to 0.32)* | | | ***-0.34 (-0.70 to 0.02) ^m^*** | | |
|  | | | | | | | | | | | | |
| **Change in total service cost (endline – baseline)** | | | | | | | | | | | | |
| - Functioning worsens | 6 | 1.61 | 7.09 | 6 | 2.65 | 6.36 | 6 | -0.93 | 1.09 | 6 | -0.10 | 0.25 |
| - Functioning improves | 9 | 22.58 | 49.40 | 9 | 22.51 | 49.68 | 9 | 0.22 | 3.19 | 9 | -0.16 | 1.22 |
| *Test of association (β, 95% CI)* | *24.14 (-11.49 to 59.77)* | | | *20.90 (-15.49 to 57.29)* | | | ***1.49 (-0.09 to 3.07) ^m^*** | | | *0.29 (-0.11 to 0.69)* | | |
|  |  |  |  |  |  |  |  |  |  |  |  |  |
| **Total private, out-of-pocket cost per 3 months** | | | |  |  |  |  |  |  |  |  |  |
| - Baseline | 39 | 4.60 | 9.16 | 39 | 3.12 | 9.07 | 39 | 0.80 | 1.95 | 39 | 0.67 | 1.88 |
| - Midline | 35 | 7.95 | 25.45 | 35 | 7.10 | 25.37 | 35 | 0.52 | 1.37 | 35 | 0.33 | 1.04 |
| - Endline | 36 | 12.09 | 27.06 | 36 | 11.16 | 27.20 | 36 | 0.65 | 1.84 | 36 | 0.28 | 0.87 |
| *Test of difference (coefficient, 95%CI)* | | | | | | | | | | | | |
| - *Midline - Baseline* | *3.16 (-5.34 to 11.67)* | | | *3.84 (-4.84 to 12.52)* | | | *-0.31 (-0.93 to 0.31)* | | | *-0.36 (-0.94 to 0.22)* | | |
| - *Endline - Baseline* | *7.41 (-1.68 to 16.50)* | | | ***7.95 (-1.02 to 16.92) ^m^*** | | | *-0.14 (-0.86 to 0.58)* | | | *-0.40 (-0.92 to 0.12)* | | |
|  | | | | | | | | | | | | |
| **Change in out-of-pocket cost (endline – baseline)** | | | | | | | | | | | | |
| - Functioning worsens | 6 | 4.39 | 14.06 | 6 | 5.78 | 13.08 | 6 | -0.74 | 1.54 | 6 | -0.65 | 1.30 |
| - Functioning improves | 9 | 24.47 | 48.27 | 9 | 23.69 | 48.79 | 9 | 0.85 | 2.98 | 9 | -0.08 | 1.30 |
| *Test of association (β, 95% CI)* | *19.29 (-21.45 to 60.03)* | | | *18.13 (-20.63 to 56.89)* | | | *1.30 (-0.97 to 3.57)* | | | ***0.47 (-0.04 to 0.99) ^m^*** | | |

^m^ marginal; * p<0.05; ** p<0.01; *** p<0.001

***Web Table 3c - Health care costs and out-of-pocket payments over time in Nepal, by disease cohort and service component (US$, 2015)***

| **Nepal - Depression** | **All care** | | | **Mental health** | | | **General** | | | **Indigenous** | | |
| --- | --- | --- | --- | --- | --- | --- | --- | --- | --- | --- | --- | --- |
|  | N | Mean | SD | N | Mean | SD | N | Mean | SD | N | Mean | SD |
| **Total service cost per 3 months** | | | | | | | | | | | | |
| - Baseline | 129 | 7.47 | 9.15 | 129 | 1.36 | 7.17 | 129 | 4.99 | 15.58 | 129 | 1.12 | 5.73 |
| - Midline | 107 | 7.50 | 19.16 | 107 | 1.43 | 1.59 | 107 | 5.67 | 18.78 | 107 | 0.39 | 1.92 |
| - Endline | 107 | 8.07 | 29.51 | 107 | 0.84 | 4.09 | 107 | 6.06 | 27.91 | 107 | 1.17 | 9.48 |
| *Test of difference (coefficient, 95%CI)* | | | | | | | | | | | | |
| - Midline - Baseline | *-0.08 (-3.55 to 3.40)* | | | *0.02 (-1.02 to 1.06)* | | | *0.78 (-2.39 to 3.95)* | | | ***-0.79 (-1.58 to -0.01) **** | | |
| - Endline - Baseline | *0.94 (-5.03 to 6.92)* | | | *-0.56 (-1.80 to 0.68)* | | | *1.73 (-3.89 to 7.35)* | | | *-0.03 (-1.94 to 1.87)* | | |
|  | | | | | | | | | | | | |
| **Change in total service cost (endline - baseline)** | | | | | | | | | | | | |
| - Functioning worsens | 24 | 17.01 | 35.61 | 24 | 0.75 | 7.05 | 24 | 11.51 | 31.08 | 24 | 4.74 | 19.75 |
| - Functioning improves | 83 | -3.30 | 33.21 | 83 | -1.06 | 9.30 | 83 | -0.65 | 29.81 | 83 | -1.59 | 7.09 |
| *Test of association (β, 95% CI)* | ***-15.23 (-30.58 to 0.13) ^m^*** | | | *-0.93 (-3.76 to 1.89)* | | | *-9.48 (-22.95 to 3.99)* | | | *-4.99 (-12.71 to 2.73)* | | |
|  |  |  |  |  |  |  |  |  |  |  |  |  |
| **Total private, out-of-pocket cost per 3 months** | | | |  |  |  |  |  |  |  |  |  |
| - Baseline | 129 | 7.66 | 18.97 | 129 | 1.40 | 7.68 | 129 | 5.12 | 15.09 | 129 | 1.14 | 5.76 |
| - Midline | 107 | 7.80 | 20.04 | 107 | 1.16 | 1.15 | 107 | 6.21 | 19.58 | 107 | 0.42 | 2.04 |
| - Endline | 107 | 8.28 | 29.57 | 107 | 0.81 | 3.86 | 107 | 6.26 | 27.98 | 107 | 1.21 | 9.58 |
| *Test of difference (coefficient, 95%CI)* | | | | | | | | | | | | |
| - Midline - Baseline | *0.01 (-3.74 to 3.75)* | | | *-0.30 (-1.41 to 0.81)* | | | *1.17 (-1.88 to 4.22)* | | | ***-0.78 (-1.57 to -0.00) **** | | |
| - Endline - Baseline | *0.97 (-5.27 to 7.22)* | | | *-0.64 (-1.96 to 0.67)* | | | *1.85 (-3.37 to 7.06)* | | | *-0.01 (-2.03 to 2.01)* | | |
|  | | | | | | | | | | | | |
| **Change in out-of-pocket cost (endline - baseline)** | | | | | | | | | | | | |
| - Functioning worsens | 24 | 16.94 | 35.43 | 24 | 0.49 | 5.80 | 24 | 11.66 | 30.88 | 24 | 4.79 | 19.95 |
| - Functioning improves | 83 | -3.26 | 32.62 | 83 | -1.09 | 10.04 | 83 | -0.60 | 28.92 | 83 | -1.57 | 7.15 |
| *Test of association (β, 95% CI)* | ***-15.44 (-30.89 to 0.02) **** | | | *-0.64 (-3.11 to 1.84)* | | | *-10.02 (-23.48 to 3.43)* | | | *-4.99 (-13.04 to 3.06)* | | |

^m^ marginal; * p<0.05; ** p<0.01; *** p<0.001

| **Nepal - AUD** | **All care** | | | **Mental health** | | | **General** | | | **Indigenous** | | |
| --- | --- | --- | --- | --- | --- | --- | --- | --- | --- | --- | --- | --- |
|  | N | Mean | SD | N | Mean | SD | N | Mean | SD | N | Mean | SD |
| **Total service cost per 3 months** | | | | | | | | | | | | |
| - Baseline | 170 | 5.06 | 17.07 | 170 | 2.28 | 14.31 | 170 | 2.33 | 9.32 | 170 | 0.44 | 2.83 |
| - Midline | 134 | 5.72 | 12.07 | 134 | 2.06 | 2.75 | 134 | 2.95 | 10.54 | 134 | 0.71 | 5.64 |
| - Endline | 140 | 12.38 | 43.85 | 140 | 0.58 | 5.21 | 140 | 10.96 | 41.36 | 140 | 0.84 | 6.82 |
| *Test of difference (coefficient, 95%CI)* | | | | | | | | | | | | |
| - Midline - Baseline | *0.40 (-2.26 to 3.06)* | | | *-0.33 (-2.19 to 1.54)* | | | *0.51 (-1.62 to 2.64)* | | | *0.37 (-0.60 to 1.34)* | | |
| - Endline - Baseline | ***7.08 (0.20 to 13.95) **** | | | ***-1.80 (-3.79 to 0.19) ^m^*** | | | ***8.59 (1.48 to 15.70) **** | | | *0.41 (-0.81 to 1.62)* | | |
|  | | | | | | | | | | | | |
| **Change in total service cost (endline - baseline)** | | | | | | | | | | | | |
| - Functioning worsens | 54 | 10.11 | 53.85 | 54 | -4.62 | 25.42 | 54 | 13.34 | 55.60 | 54 | 1.38 | 11.81 |
| - Functioning improves | 86 | 4.96 | 31.97 | 86 | -0.34 | 6.64 | 86 | 5.49 | 31.39 | 86 | -0.19 | 1.63 |
| *Test of association (β, 95% CI)* | *-6.65 (-22.82 to 9.51)* | | | *0.31 (-1.16 to 1.77)* | | | *-9.15 (-25.61 to 7.32)* | | | *-2.14 (-5.08 to 0.79)* | | |
|  |  |  |  |  |  |  |  |  |  |  |  |  |
| **Total private, out-of-pocket cost per 3 months** | | | |  |  |  |  |  |  |  |  |  |
| - Baseline | 170 | 5.03 | 16.70 | 170 | 2.31 | 14.41 | 170 | 2.25 | 8.44 | 170 | 0.46 | 3.03 |
| - Midline | 134 | 5.35 | 11.72 | 134 | 1.70 | 1.75 | 134 | 2.80 | 9.34 | 134 | 0.85 | 6.97 |
| - Endline | 140 | 12.58 | 45.14 | 140 | 0.58 | 5.21 | 140 | 11.08 | 42.15 | 140 | 0.92 | 7.64 |
| *Test of difference (coefficient, 95%CI)* | | | | | | | | | | | | |
| - Midline - Baseline | *-0.00 (-2.46 to 2.45)* | | | *-0.72 (-2.50 to 1.06)* | | | *0.40 (-1.61 to 2.40)* | | | *0.50 (-0.68 to 1.67)* | | |
| - Endline - Baseline | ***7.23 (0.21 to 14.24) **** | | | ***-1.83 (-3.84 to 0.19) ^m^*** | | | ***8.76 (1.54 to 15.98) **** | | | *0.46 (-0.80 to 1.73)* | | |
|  | | | | | | | | | | | | |
| **Change in out-of-pocket cost (endline - baseline)** | | | | | | | | | | | | |
| - Functioning worsens | 54 | 10.85 | 54.30 | 54 | -4.72 | 25.58 | 54 | 14.04 | 56.26 | 54 | 1.53 | 13.17 |
| - Functioning improves | 86 | 4.76 | 31.72 | 86 | -0.33 | 6.64 | 86 | 5.28 | 31.13 | 86 | -0.19 | 1.63 |
| *Test of association (β, 95% CI)* | *-6.61 (-22.74 to 9.53)* | | | *0.30 (-1.13 to 1.73)* | | | *-9.53 (-25.98 to 6.93)* | | | *-2.35 (-5.67 to 0.97)* | | |

^m^ marginal; * p<0.05; ** p<0.01; *** p<0.001

| **Nepal – psychosis** | **All care** | | | **Mental health** | | | **General** | | | **Indigenous** | | |
| --- | --- | --- | --- | --- | --- | --- | --- | --- | --- | --- | --- | --- |
|  | N | Mean | SD | N | Mean | SD | N | Mean | SD | N | Mean | SD |
| **Total service cost per 3 months** | | | | | | | | | | | | |
| - Baseline | 93 | 18.52 | 40.61 | 93 | 11.76 | 28.56 | 93 | 4.27 | 18.24 | 93 | 3.48 | 22.70 |
| - Midline | 87 | 10.86 | 20.29 | 87 | 8.70 | 20.02 | 87 | 2.05 | 5.26 | 87 | 0.11 | 0.77 |
| - Endline | 86 | 13.39 | 29.99 | 86 | 6.04 | 13.31 | 86 | 7.29 | 25.72 | 86 | 0.06 | 0.57 |
| *Test of difference (coefficient, 95%CI)* | | | | | | | | | | | | |
| - Midline - Baseline | ***-8.36 (-15.19 to -1.52) **** | | | *-3.20 (-9.36 to 2.96)* | | | *-1.68 (-4.28 to 0.92)* | | | ***-3.45 (-7.01 to 0.10) ^m^*** | | |
| - Endline - Baseline | *-6.56 (-15.05 to 1.93)* | | | ***-5.90 (-10.53 to -1.26) **** | | | *2.87 (-3.46 to 9.20)* | | | ***-3.51 (-7.12 to 0.10) ^m^*** | | |
|  | | | | | | | | | | | | |
| **Change in total service cost (endline - baseline)** | | | | | | | | | | | | |
| - Functioning worsens | 25 | -3.16 | 49.19 | 25 | 0.76 | 19.10 | 25 | -2.95 | 43.79 | 25 | -0.97 | 4.85 |
| - Functioning improves | 61 | -8.85 | 48.14 | 61 | -9.00 | 29.72 | 61 | 4.97 | 26.24 | 61 | -4.82 | 27.86 |
| *Test of association (β, 95% CI)* | *-4.72 (-19.41 to 9.96)* | | | *-4.67 (-12.44 to 3.10)* | | | *-1.51 (-13.84 to 10.83)* | | | *0.09 (-0.07 to 0.25)* | | |
|  |  |  |  |  |  |  |  |  |  |  |  |  |
| **Total private, out-of-pocket cost per 3 months** | | | |  |  |  |  |  |  |  |  |  |
| - Baseline | 93 | 20.46 | 41.94 | 93 | 12.20 | 28.64 | 93 | 4.44 | 18.33 | 93 | 3.82 | 24.01 |
| - Midline | 87 | 12.95 | 22.21 | 87 | 9.45 | 21.50 | 87 | 3.11 | 7.06 | 87 | 0.38 | 3.33 |
| - Endline | 86 | 14.12 | 29.82 | 86 | 6.00 | 12.86 | 86 | 8.01 | 26.94 | 86 | 0.11 | 0.99 |
| *Test of difference (coefficient, 95%CI)* | | | | | | | | | | | | |
| - Midline - Baseline | *-7.22 (-14.39 to -0.06) ** | | | *-2.89 (-8.82 to 3.06)* | | | *-0.80 (-3.62 to 2.01)* | | | ***-3.52 (-7.59 to 0.55) ^m^*** | | |
| - Endline - Baseline | *-6.78 (-15.28 to 1.72)* | | | ***-6.34 (-10.61 to -2.07) ***** | | | *3.42 (-3.30 to 10.15)* | | | ***-3.81 (-7.91 to 0.29) ^m^*** | | |
|  | | | | | | | | | | | | |
| **Change in out-of-pocket cost (endline - baseline)** | | | | | | | | | | | | |
| - Functioning worsens | 25 | -4.22 | 48.76 | 25 | -0.33 | 17.24 | 25 | -2.68 | 44.07 | 25 | -1.20 | 6.01 |
| - Functioning improves | 61 | -8.65 | 49.92 | 61 | -9.08 | 29.77 | 61 | 5.62 | 27.90 | 61 | -5.17 | 29.42 |
| *Test of association (β, 95% CI)* | *-3.65 (-17.64 to 10.34)* | | | *-4.08 (-11.07 to 2.92)* | | | *-1.07 (-14.22 to 12.08)* | | | *0.15 (-0.13 to 0.44)* | | |

^m^ marginal; * p<0.05; ** p<0.01; *** p<0.001

| **Nepal – epilepsy** | **All care** | | | **Mental health** | | | **General** | | | **Indigenous** | | |
| --- | --- | --- | --- | --- | --- | --- | --- | --- | --- | --- | --- | --- |
|  | N | Mean | SD | N | Mean | SD | N | Mean | SD | N | Mean | SD |
| **Total service cost per 3 months** | | | | | | | | | | | | |
| - Baseline | 41 | 18.67 | 55.49 | 41 | 11.28 | 49.02 | 41 | 7.23 | 28.77 | 41 | 0.17 | 1.06 |
| - Midline | 39 | 3.70 | 4.05 | 39 | 2.59 | 2.34 | 39 | 1.11 | 3.35 | 39 | 0.00 | 0.00 |
| - Endline | 37 | 10.58 | 29.91 | 37 | 3.70 | 7.00 | 37 | 2.22 | 7.57 | 37 | 4.66 | 27.07 |
| *Test of difference (coefficient, 95%CI)* | | | | | | | | | | | | |
| - Midline - Baseline | ***-15.13 (-28.12 to -2.13) **** | | | *-8.84 (-19.85 to 2.17)* | | | ***-6.12 (-12.83 to 0.59) ^m^*** | | | *-0.17 (-0.50 to 0.17)* | | |
| - Endline - Baseline | *-8.36 (-25.62 to 8.91)* | | | *-7.77 (-18.84 to 3.31)* | | | *-5.08 (-12.67 to 2.51)* | | | *4.49 (-3.84 to 12.83)* | | |
|  | | | | | | | | | | | | |
| **Change in total service cost (endline - baseline)** | | | | | | | | | | | | |
| - Functioning worsens | 11 | -12.92 | 59.27 | 11 | 3.75 | 12.55 | 11 | -17.12 | 57.00 | 11 | 0.44 | 1.18 |
| - Functioning improves | 26 | -7.16 | 70.59 | 26 | -13.16 | 61.18 | 26 | -0.19 | 3.07 | 26 | 6.18 | 32.37 |
| *Test of association (β, 95% CI)* | *-0.91 (-17.00 to 15.17)* | | | *-4.38 (-11.82 to 3.06)* | | | *-2.63 (-11.20 to 5.94)* | | | *5.74 (-6.03 to 17.51)* | | |
|  |  |  |  |  |  |  |  |  |  |  |  |  |
| **Total private, out-of-pocket cost per 3 months** | | | |  |  |  |  |  |  |  |  |  |
| - Baseline | 41 | 18.74 | 57.24 | 41 | 12.09 | 53.24 | 41 | 6.48 | 24.24 | 41 | 0.17 | 1.06 |
| - Midline | 39 | 4.26 | 5.91 | 39 | 3.16 | 4.94 | 39 | 1.10 | 3.48 | 39 | 0.00 | 0.00 |
| - Endline | 37 | 12.17 | 35.92 | 37 | 4.41 | 8.36 | 37 | 1.78 | 7.15 | 37 | 5.76 | 33.44 |
| *Test of difference (coefficient, 95%CI)* | | | | | | | | | | | | |
| - Midline - Baseline | ***-14.62 (-28.23 to -1.01) **** | | | *-9.09 (-21.33 to 3.15)* | | | ***-5.36 (-10.74 to 0.02) ^m^*** | | | *-0.17 (-0.49 to 0.16)* | | |
| - Endline - Baseline | *-6.82 (-24.33 to 10.69)* | | | *-7.89 (-20.81 to 5.03)* | | | *-4.73 (-11.27 to 1.81)* | | | *5.59 (-4.92 to 16.10)* | | |
|  | | | | | | | | | | | | |
| **Change in OOP cost (EL-BL)** | | | | | | | | | | | | |
| - Functioning worsens | 11 | -7.77 | 54.36 | 11 | 5.45 | 14.24 | 11 | -13.82 | 48.95 | 11 | 0.62 | 1.75 |
| - Functioning improves | 26 | -7.13 | 79.15 | 26 | -14.12 | 66.42 | 26 | -0.97 | 5.06 | 26 | 7.67 | 39.97 |
| *Test of association (β, 95% CI)* | *-1.94 (-23.88 to 20.00)* | | | *-5.97 (-14.20 to 2.26)* | | | *-4.23 (-13.24 to 4.79)* | | | *7.06 (-8.30 to 22.41)* | | |

^m^ marginal; * p<0.05; ** p<0.01; *** p<0.001

***Web Table 3d - Health care costs and out-of-pocket payments over time in South Africa, by disease cohort and service component (US$, 2015)***

| **SA - Depression** | **All care** | | | **Mental health** | | | **General** | | | **Indigenous** | | |
| --- | --- | --- | --- | --- | --- | --- | --- | --- | --- | --- | --- | --- |
|  | N | Mean | SD | N | Mean | SD | N | Mean | SD | N | Mean | SD |
| **Total service cost per 3 months** | | | | | | | | | | | | |
| - Baseline | 216 | 19.30 | 29.86 | 216 | 1.98 | 7.59 | 216 | 15.89 | 27.89 | 216 | 1.43 | 6.37 |
| - Midline | 192 | 47.61 | 66.45 | 192 | 32.01 | 60.33 | 192 | 14.36 | 25.77 | 192 | 1.23 | 6.63 |
| - Endline | 176 | 16.70 | 28.34 | 176 | 1.41 | 7.40 | 176 | 14.55 | 25.89 | 176 | 0.73 | 4.57 |
| *Test of difference (coefficient, 95%CI)* | | | | | | | | | | | | |
| - Midline - Baseline | ***27.69 (17.95 to 37.44) ****** | | | ***29.96 (21.56 to 38.36) ****** | | | *-2.04 (-6.03 to 1.95)* | | | *-0.24 (-1.33 to 0.86)* | | |
| - Endline - Baseline | *-3.16 (-8.04 to 1.72)* | | | *-0.37 (-1.62 to 0.87)* | | | *-1.98 (-6.66 to 2.70)* | | | *-0.75 (-1.69 to 0.18)* | | |
|  | | | | | | | | | | | | |
| **Change in total service cost (endline - baseline)** | | | | | | | | | | | | |
| - Functioning worsens | 65 | -0.58 | 53.90 | 65 | 1.94 | 11.31 | 65 | -1.85 | 51.39 | 65 | -0.66 | 6.82 |
| - Functioning improves | 111 | -5.70 | 31.09 | 111 | -1.53 | 7.49 | 111 | -3.22 | 29.19 | 111 | -0.94 | 8.46 |
| *Test of association (β, 95% CI)* | ***-9.57 (-19.66 to 0.53) ^m^*** | | | *-2.06 (-4.84 to 0.71)* | | | ***-7.90 (-16.82 to 1.03) ^m^*** | | | *0.22 (-1.19 to 1.63)* | | |
|  |  |  |  |  |  |  |  |  |  |  |  |  |
| **Total private, out-of-pocket cost per 3 months** | | | |  |  |  |  |  |  |  |  |  |
| - Baseline | 216 | 5.52 | 12.16 | 216 | 0.45 | 2.56 | 216 | 3.37 | 7.95 | 216 | 1.70 | 7.28 |
| - Midline | 192 | 5.63 | 11.86 | 192 | 0.65 | 2.55 | 192 | 3.68 | 8.93 | 192 | 1.30 | 6.93 |
| - Endline | 176 | 4.84 | 8.95 | 176 | 0.33 | 1.90 | 176 | 3.61 | 6.88 | 176 | 0.90 | 5.30 |
| *Test of difference (coefficient, 95%CI)* | | | | | | | | | | | | |
| - Midline - Baseline | *-0.06 (-2.07 to 1.95)* | | | *0.17 (-0.22 to 0.55)* | | | *0.22 (-1.18 to 1.61)* | | | *-0.45 (-1.57 to 0.66)* | | |
| - Endline - Baseline | *-0.89 (-2.65 to 0.87)* | | | *-0.17 (-0.46 to 0.13)* | | | *0.14 (-1.07 to 1.35)* | | | ***-0.87 (-1.88 to 0.13) ^m^*** | | |
|  | | | | | | | | | | | | |
| **Change in out-of-pocket cost (endline - baseline)** | | | | | | | | | | | | |
| - Functioning worsens | 65 | 0.42 | 15.66 | 65 | 0.09 | 1.82 | 65 | 1.42 | 10.97 | 65 | -1.09 | 9.34 |
| - Functioning improves | 111 | -2.09 | 14.82 | 111 | -0.38 | 2.87 | 111 | -0.76 | 8.87 | 111 | -0.95 | 9.20 |
| *Test of association (β, 95% CI)* | ***-2.92 (-5.87 to 0.04) ^m^*** | | | *-0.32 (-0.83 to 0.19)* | | | ***-2.66 (-4.89 to -0.42) **** | | | *0.06 (-1.59 to 1.71)* | | |

^m^ marginal; * p<0.05; ** p<0.01; *** p<0.001

| **SA - Psychosis** | **All care** | | | **Mental health** | | | **General** | | | **Indigenous** | | |
| --- | --- | --- | --- | --- | --- | --- | --- | --- | --- | --- | --- | --- |
|  | N | Mean | SD | N | Mean | SD | N | Mean | SD | N | Mean | SD |
| **Total service cost per 3 months** | | | | | | | | | | | | |
| - Baseline | 29 | 50.65 | 84.03 | 29 | 35.42 | 75.47 | 29 | 14.62 | 24.95 | 29 | 0.61 | 3.04 |
| - Midline | 2 | 0.00 | 0.00 | 2 | 0.00 | 0.00 | 2 | 0.00 | 0.00 | 2 | 0.00 | 0.00 |
| - Endline | 7 | 33.89 | 67.09 | 7 | 25.74 | 68.10 | 7 | 8.15 | 6.74 | 7 | 0.00 | 0.00 |
| *Test of difference (coefficient, 95%CI)^1^* | | | | | | | | | | | | |
| - Midline - Baseline |  | | |  | | |  | | |  | | |
| - Endline - Baseline |  | | |  | | |  | | |  | | |
|  | | | | | | | | | | | | |
| **Change in total service cost (endline - baseline)** | | | | | | | | | | | | |
| - Functioning worsens | 1 | 7.08 | - | 1 | -7.78 | - | 1 | 14.86 | - | 1 | 0.00 | - |
| - Functioning improves | 6 | 10.49 | 96.82 | 6 | 25.81 | 76.30 | 6 | -15.32 | 46.87 | 6 | 0.00 | 0.00 |
| *Test of association (β, 95% CI)^1^* | ***-*** | | | *-* | | | *-* | | | *-* | | |
|  |  |  |  |  |  |  |  |  |  |  |  |  |
| **Total private, out-of-pocket cost per 3 months** | | | |  |  |  |  |  |  |  |  |  |
| - Baseline | 29 | 15.32 | 16.67 | 29 | 13.89 | 16.43 | 29 | 0.81 | 2.29 | 29 | 0.61 | 3.04 |
| - Midline | 2 | 0.00 | 0.00 | 2 | 0.00 | 0.00 | 2 | 0.00 | 0.00 | 2 | 0.00 | 0.00 |
| - Endline | 7 | 7.97 | 14.30 | 7 | 5.42 | 14.35 | 7 | 2.55 | 5.54 | 7 | 0.00 | 0.00 |
| *Test of difference (coefficient, 95%CI)^1^* | | | | | | | | | | | | |
| - Midline - Baseline |  | | |  | | |  | | |  | | |
| - Endline - Baseline |  | | |  | | |  | | |  | | |
|  | | | | | | | | | | | | |
| **Change in out-of-pocket cost (endline - baseline)** | | | | | | | | | | | | |
| - Functioning worsens | 1 | 7.08 | - | 1 | -7.78 | - | 1 | 14.86 | - | 1 | 0.00 | - |
| - Functioning improves | 6 | 2.61 | 20.25 | 6 | 2.11 | 20.27 | 6 | 0.50 | 1.21 | 6 | 0.00 | 0.00 |
| *Test of association (β, 95% CI)^1^* | *-* | | | *-* | | | *-* | | | *-* | | |

^1^ Sample size too small to allow tests of difference or association

***Web Table 3e - Health care costs and out-of-pocket payments over time in Uganda, by disease cohort and service component (US$, 2015)***

| **Uganda - Depression** | **All care** | | | **Mental health** | | | **General** | | | **Indigenous** | | |
| --- | --- | --- | --- | --- | --- | --- | --- | --- | --- | --- | --- | --- |
|  | N | Mean | SD | N | Mean | SD | N | Mean | SD | N | Mean | SD |
| **Total service cost per 3 months** | | | | | | | | | | | | |
| - Baseline | 63 | 6.69 | 8.51 | 63 | 1.75 | 2.10 | 63 | 4.64 | 8.00 | 63 | 0.30 | 1.91 |
| - Midline | 60 | 6.44 | 8.30 | 60 | 1.94 | 2.37 | 60 | 3.29 | 4.86 | 60 | 1.20 | 5.48 |
| - Endline | 56 | 3.28 | 6.64 | 56 | 0.41 | 0.99 | 56 | 2.45 | 5.41 | 56 | 0.43 | 2.32 |
| *Test of difference (coefficient, 95%CI)* | | | | | | | | | | | | |
| - Midline - Baseline | *-0.37 (-2.76 to 2.02)* | | | *0.14 (-0.43 to 0.71)* | | | *-1.41 (-3.27 to 0.44)* | | | *0.90 (-0.61 to 2.40)* | | |
| - Endline - Baseline | ***-3.23 (-5.51 to -0.94) ***** | | | ***-1.32 (-1.69 to -0.95) ****** | | | ***-2.03 (-4.06 to 0.01) **** | | | *0.12 (-0.59 to 0.83)* | | |
|  | | | | | | | | | | | | |
| **Change in total service cost (endline - baseline)** | | | | | | | | | | | | |
| - Functioning worsens | 11 | 2.23 | 5.90 | 11 | -1.32 | 1.48 | 11 | 2.19 | 5.61 | 11 | 1.37 | 4.53 |
| - Functioning improves | 45 | -4.28 | 10.59 | 45 | -1.29 | 1.82 | 45 | -2.77 | 9.20 | 45 | -0.22 | 2.66 |
| *Test of association (β, 95% CI)* | *-3.24 (-7.64 to 1.16)* | | | *0.18 (-0.25 to 0.62)* | | | *-2.23 (-5.63 to 1.17)* | | | *-1.16 (-3.86 to 1.54)* | | |
|  |  |  |  |  |  |  |  |  |  |  |  |  |
| **Total private, out-of-pocket cost per 3 months** | | | |  |  |  |  |  |  |  |  |  |
| - Baseline | 63 | 5.53 | 7.64 | 63 | 1.50 | 1.78 | 63 | 3.56 | 6.87 | 63 | 0.47 | 2.75 |
| - Midline | 60 | 6.81 | 9.89 | 60 | 7.73 | 2.13 | 60 | 3.41 | 5.80 | 60 | 1.67 | 6.07 |
| - Endline | 56 | 4.24 | 8.48 | 56 | 0.43 | 1.14 | 56 | 3.10 | 6.58 | 56 | 0.71 | 3.16 |
| *Test of difference (coefficient, 95%CI)* | | | | | | | | | | | | |
| - Midline - Baseline | *1.20 (-1.48 to 3.88)* | | | *0.19 (-0.37 to 0.75)* | | | *-0.19 (-2.11 to 1.74)* | | | *1.20 (-0.43 to 2.82)* | | |
| - Endline - Baseline | *-1.31 (-3.80 to 1.19)* | | | ***-1.12 (-1.45 to -0.78) ****** | | | *-0.42 (-2.45 to 1.61)* | | | *0.23 (-0.75 to 1.21)* | | |
|  | | | | | | | | | | | | |
| **Change in out-of-pocket cost (endline - baseline)** | | | | | | | | | | | | |
| - Functioning worsens | 11 | 3.51 | 6.95 | 11 | -1.32 | 1.48 | 11 | 2.43 | 5.40 | 11 | 2.40 | 5.76 |
| - Functioning improves | 45 | -2.51 | 11.39 | 45 | -1.10 | 1.58 | 45 | -1.05 | 9.35 | 45 | -0.35 | 3.87 |
| *Test of association (β, 95% CI)* | *-3.75 (-8.28 to 0.79)* | | | *0.24 (-0.23 to 0.71)* | | | *-1.83 (-5.26 to 1.60)* | | | *-2.09 (-5.64 to 1.46)* | | |

^m^ marginal; * p<0.05; ** p<0.01; *** p<0.001

| **Uganda - Psychosis** | **All care** | | | **Mental health** | | | **General** | | | **Indigenous** | | |
| --- | --- | --- | --- | --- | --- | --- | --- | --- | --- | --- | --- | --- |
|  | N | Mean | SD | N | Mean | SD | N | Mean | SD | N | Mean | SD |
| **Total service cost per 3 months** | | | | | | | | | | | | |
| - Baseline | 51 | 12.30 | 19.49 | 51 | 8.67 | 15.04 | 51 | 0.96 | 2.38 | 51 | 2.67 | 13.62 |
| - Midline | 48 | 7.07 | 8.66 | 48 | 5.91 | 7.64 | 48 | 1.16 | 3.54 | 48 | 0.00 | 0.00 |
| - Endline | 43 | 4.84 | 6.27 | 43 | 3.41 | 4.89 | 43 | 1.43 | 4.35 | 43 | 0.00 | 0.00 |
| *Test of difference (coefficient, 95%CI)* | | | | | | | | | | | | |
| - Midline - Baseline | ***-5.16 (-9.60 to -0.72) **** | | | *-2.65 (-4.04 to 0.74)* | | | *0.17 (-0.84 to 1.18)* | | | ***-2.68 (-5.50 to 0.13) ^m^*** | | |
| - Endline - Baseline | ***-7.37 (-11.87 to -2.88) ***** | | | ***-4.94 (-8.19 to -1.70) ***** | | | *0.35 (-0.63 to 1.34)* | | | ***-2.79 (-5.89 to 0.31) ^m^*** | | |
|  | | | | | | | | | | | | |
| **Change in total service cost (endline - baseline)** | | | | | | | | | | | | |
| - Functioning worsens | 13 | -3.46 | 9.63 | 13 | -4.24 | 8.89 | 13 | 0.90 | 2.60 | 13 | -0.11 | 0.42 |
| - Functioning improves | 30 | -8.87 | 24.56 | 30 | -4.65 | 16.85 | 30 | 0.06 | 3.84 | 30 | -4.29 | 17.67 |
| *Test of association (β, 95% CI)* | *0.26 (-4.07 to 4.58)* | | | *1.26 (-1.44 to 3.96)* | | | *-0.83 (-3.09 to 1.43)* | | | *-4.17 (-10.36 to 2.02)* | | |
|  |  |  |  |  |  |  |  |  |  |  |  |  |
| **Total private, out-of-pocket cost per 3 months** | | | |  |  |  |  |  |  |  |  |  |
| - Baseline | 51 | 9.00 | 17.68 | 51 | 4.16 | 5.71 | 51 | 0.88 | 2.42 | 51 | 3.96 | 16.14 |
| - Midline | 48 | 4.01 | 5.96 | 48 | 2.73 | 2.61 | 48 | 1.27 | 4.78 | 48 | 0.01 | 0.04 |
| - Endline | 43 | 3.91 | 4.40 | 43 | 2.90 | 3.42 | 43 | 1.01 | 2.99 | 43 | 0.00 | 0.00 |
| *Test of difference (coefficient, 95%CI)* | | | | | | | | | | | | |
| - Midline - Baseline | ***-5.06 (-9.08 to -1.05) **** | | | ***-1.51 (-2.90 to -0.12) **** | | | *0.36 (-1.03 to 1.74)* | | | ***-3.93 (-7.37 to -0.50) **** | | |
| - Endline - Baseline | ***-5.09 (-9.18 to -1.00) **** | | | ***-1.29 (-2.72 to 0.15) ^m^*** | | | *0.04 (-0.74 to 0.83)* | | | ***-3.88 (-7.39 to -0.38) **** | | |
|  | | | | | | | | | | | | |
| **Change in out-of-pocket cost (endline - baseline)** | | | | | | | | | | | | |
| - Functioning worsens | 13 | -2.06 | 5.08 | 13 | -1.71 | 3.30 | 13 | -0.24 | 3.29 | 13 | -0.12 | 0.42 |
| - Functioning improves | 30 | -6.40 | 22.27 | 30 | -1.17 | 6.29 | 30 | 0.07 | 2.56 | 30 | -5.31 | 20.40 |
| *Test of association (β, 95% CI)* | *1.41 (-1.07 to 3.89)* | | | *1.08 (-0.55 to 2.71)* | | | *0.25 (-1.32 to 1.82)* | | | *-5.19 (-12.17 to 1.79)* | | |

^m^ marginal; * p<0.05; ** p<0.01

| **Uganda - Epilepsy** | **All care** | | | **Mental health** | | | **General** | | | **Indigenous** | | |
| --- | --- | --- | --- | --- | --- | --- | --- | --- | --- | --- | --- | --- |
|  | N | Mean | SD | N | Mean | SD | N | Mean | SD | N | Mean | SD |
| **Total service cost per 3 months** | | | | | | | | | | | | |
| - Baseline | 181 | 5.12 | 7.13 | 181 | 2.79 | 3.25 | 181 | 1.10 | 4.48 | 181 | 1.24 | 4.83 |
| - Midline | 174 | 4.79 | 6.47 | 174 | 3.16 | 3.37 | 174 | 0.89 | 2.70 | 174 | 0.74 | 4.37 |
| - Endline | 160 | 3.36 | 4.23 | 160 | 2.31 | 2.01 | 160 | 0.54 | 1.62 | 160 | 0.52 | 3.56 |
| *Test of difference (coefficient, 95%CI)* | | | | | | | | | | | | |
| - Midline - Baseline | *-0.30 (-1.41 to 0.82)* | | | *0.41 (-0.14 to 0.96)* | | | *-0.23 (-0.75 to 0.29)* | | | *-0.48 (-1.33 to 0.37)* | | |
| - Endline - Baseline | ***-1.75 (-2.75 to -0.75) ***** | | | ***-0.45 (-0.87 to -0.03) **** | | | ***-0.59 (-1.11 to -0.06) **** | | | ***-0.72 (-1.46 to 0.02) ^m^*** | | |
|  | | | | | | | | | | | | |
| **Change in total service cost (endline - baseline)** | | | | | | | | | | | | |
| - Functioning worsens | 70 | -1.43 | 7.91 | 70 | -0.93 | 3.54 | 70 | -0.16 | 1.69 | 70 | -0.33 | 7.41 |
| - Functioning improves | 89 | -1.95 | 8.25 | 89 | 0.03 | 3.19 | 89 | 0.99 | 6.28 | 89 | -1.02 | 3.76 |
| *Test of association (β, 95% CI)* | *-0.07 (-1.47 to 1.33)* | | | ***0.57 (-0.02 to 1.16) ^m^*** | | | *-0.14 (-0.70 to 0.42)* | | | *-0.47 (-1.71 to 0.76)* | | |
|  |  |  |  |  |  |  |  |  |  |  |  |  |
| **Total private, out-of-pocket cost per 3 months** | | | |  |  |  |  |  |  |  |  |  |
| - Baseline | 181 | 4.71 | 7.60 | 181 | 2.41 | 2.84 | 181 | 0.58 | 2.00 | 181 | 1.74 | 7.04 |
| - Midline | 174 | 4.44 | 6.45 | 174 | 2.76 | 3.03 | 174 | 0.79 | 2.64 | 174 | 0.87 | 4.81 |
| - Endline | 160 | 3.87 | 5.49 | 160 | 2.42 | 2.34 | 160 | 0.64 | 1.82 | 160 | 0.79 | 4.84 |
| *Test of difference (coefficient, 95%CI)* | | | | | | | | | | | | |
| - Midline - Baseline | *-0.29 (-1.42 to 0.84)* | | | *0.34 (-0.14 to 0.81)* | | | *0.20 (-0.14 to 0.54)* | | | *-0.85 (-1.90 to 0.19)* | | |
| - Endline - Baseline | *-0.87 (-2.01 to 0.27)* | | | *0.04 (-0.40 to 0.47)* | | | *0.05 (-0.24 to 0.34)* | | | ***-0.96 (-2.04 to 0.11) ^m^*** | | |
|  | | | | | | | | | | | | |
| **Change in out-of-pocket cost (endline - baseline)** | | | | | | | | | | | | |
| - Functioning worsens | 70 | -0.62 | 8.28 | 70 | -0.38 | 3.13 | 70 | 0.11 | 1.34 | 70 | -0.37 | 8.08 |
| - Functioning improves | 89 | -1.08 | 9.61 | 89 | 0.41 | 2.87 | 89 | -0.01 | 2.85 | 89 | -1.50 | 8.34 |
| *Test of association (β, 95% CI)* | *0.37 (-1.39 to 2.13)* | | | ***0.73 (0.04 to 1.42) **** | | | *0.00 (-0.57 to 0.57)* | | | *-0.38 (-1.84 to 1.09)* | | |

^m^ marginal; * p<0.05; ** p<0.01; *** p<0.001
