# Supplementary material for: Health service costs and their association with functional impairment among adults receiving integrated mental health care in five low- and middle-income countries: the PRIME cohort study
Source: Health Policy Plan. 2020 Mar 9;35(5):567–76. doi: 10.1093/heapol/czz182 (PMC11318687; doi:10.1093/heapol/czz182)
Supplement: czz182_Supplementary_Data [file heapol_35_5_567_s1.zip › czz182-Suppl_Data/Web Appendix 1 Unit costs of health services and goods.docx]

**Web Appendix 1 Unit costs of health services and goods (US$, 2015)**

|  | *Unit* | **Ethiopia** | **India** | **Nepal** | **S Africa** | **Uganda** |
| --- | --- | --- | --- | --- | --- | --- |
|  |  |  |  |  |  |  |
| **US$ exchange rate (2015)** |  | 20.5 | 64 | 101 | 12.7 | 3202 |
| **Inpatient care** |  |  |  |  |  |  |
| Private hospital | *Day* | $ 2.47 | $ 12.68 | $ 5.26 | $ 30.02 | $ 4.17 |
| Public / NGO hospital | *Day* | $ 1.92 | $ 9.85 | $ 4.08 | $ 21.05 | $ 3.23 |
|  |  |  |  |  |  |  |
| **Outpatient care** |  |  |  |  |  |  |
| Private hospital | *Visit* | $ 1.14 | $ 3.94 | $ 2.08 | $ 5.28 | $ 1.67 |
| Public / NGO hospital | *Visit* | $ 0.81 | $ 2.79 | $ 1.47 | $ 4.09 | $ 1.19 |
|  |  |  |  |  |  |  |
| Specialist doctor / psychiatrist |  |  |  |  |  |  |
| Consultation in private hospital | *Minute* | $ 0.17 | $ 0.59 | $ 0.31 | $ 0.79 | $ 0.25 |
| Consultation in public / NGO hospital | *Minute* | $ 0.12 | $ 0.42 | $ 0.22 | $ 0.61 | $ 0.18 |
|  |  |  |  |  |  |  |
| General doctor / medical officer |  |  |  |  |  |  |
| Consultation in private hospital | *Minute* | $ 0.14 | $ 0.49 | $ 0.26 | $ 0.66 | $ 0.21 |
| Consultation in public / NGO hospital | *Minute* | $ 0.10 | $ 0.35 | $ 0.18 | $ 0.51 | $ 0.15 |
| Consultation outside hospital (e.g. health centre) | *Minute* | $ 0.04 | $ 0.13 | $ 0.05 | $ 0.54 | $ 0.06 |
|  |  |  |  |  |  |  |
| Nurse / midwife / psychiatric nurse |  |  |  |  |  |  |
| Consultation in private hospital | *Minute* | $ 0.04 | $ 0.15 | $ 0.08 | $ 0.20 | $ 0.06 |
| Consultation in public / NGO hospital | *Minute* | $ 0.03 | $ 0.10 | $ 0.06 | $ 0.15 | $ 0.04 |
| Consultation outside hospital (e.g. health centre) | *Minute* | $ 0.03 | $ 0.05 | $ 0.03 | $ 0.21 | $ 0.04 |
|  |  |  |  |  |  |  |
| Counsellor / social worker |  |  |  |  |  |  |
| Consultation in private hospital | *Minute* | $ 0.04 | $ 0.15 | $ 0.08 | $ 0.20 | $ 0.06 |
| Consultation in public / NGO hospital | *Minute* | $ 0.03 | $ 0.10 | $ 0.06 | $ 0.15 | $ 0.04 |
| Consultation outside hospital (e.g. health centre) | *Minute* | $ 0.03 | $ 0.05 | $ 0.03 | $ 0.21 | $ 0.04 |
|  |  |  |  |  |  |  |
| Community health worker |  |  |  |  |  |  |
| Consultation outside hospital (e.g. health centre) | *Minute* | $ 0.02 | $ 0.01 | $ 0.004 | $ 0.05 | $ - |
|  |  |  |  |  |  |  |
| **Psychological treatment** |  |  |  |  |  |  |
| Individual session | *Minute* | $ 0.03 | $ 0.05 | $ 0.03 | $ 0.21 | $ 0.04 |
| Group session (10 persons, 2 providers assumed) | *Minute* | $ 0.01 | $ 0.01 | $ 0.01 | $ 0.04 | $ 0.01 |
|  |  |  |  |  |  |  |
| **Psychotropic medication** |  |  |  |  |  |  |
| Anti-psychotic medication |  |  |  |  |  |  |
| Chlopromazine (100 mg) | *Tablet* | $ 0.020 | $ 0.014 | $ 0.020 | $ 0.208 | $ 0.014 |
| Haloperidol (5 mg) | *Tablet* | $ 0.040 | $ 0.004 | $ 0.030 | $ 0.286 | $ 0.004 |
| Risperidone (2 mg) | *Tablet* | $ 0.088 | $ 0.088 | $ 0.030 | $ 1.142 | $ 0.088 |
| Olanzapine | *Tablet* | $ - | $ - | $ - | $ - | $ - |
| Fluphenazine (25 mg) | *Tablet* | $ 0.810 | $ 0.810 | $ 0.810 | $ 25.433 | $ 0.810 |
| Mood-stabliser medication |  |  |  |  |  |  |
| Carbemazepine (200 mg) | *Tablet* | $ 0.020 | $ 0.020 | $ 0.020 | $ 0.233 | $ 0.020 |
| Valproate (1500 mg) | *Tablet* | $ 0.203 | $ 0.203 | $ 0.203 | $ 0.072 | $ 0.203 |
| Anti-depressant medication |  |  |  |  |  |  |
| Amytryptyline (50 mg) | *Tablet* | $ 0.030 | $ 0.016 | $ 0.059 | $ 0.061 | $ 0.016 |
| Imipramine (100 mg) | *Tablet* | $ 0.016 | $ 0.016 | $ 0.016 | $ 0.016 | $ 0.016 |
| Fluoxetine (20 mg) | *Tablet* | $ 0.027 | $ 0.027 | $ 0.069 | $ 0.090 | $ 0.027 |
| Anti-epileptic medication |  |  |  |  |  |  |
| Phenobarbitone (100 mg) | *Tablet* | $ 0.007 | $ 0.007 | $ 0.030 | $ 0.011 | $ 0.007 |
| Sodium valproate (200 mg) | *Tablet* | $ 0.068 | $ 0.068 | $ 0.069 | $ 0.072 | $ 0.068 |
| Phenytoin (100 mg) | *Tablet* | $ 0.004 | $ 0.004 | $ 0.040 | $ 0.159 | $ 0.004 |
| Lamotrigine | *Tablet* | $ - | $ - | $ - | $ - | $ - |
| Other medications |  | $ - | $ - | $ - | $ - | $ - |
| Thiamine (100 mg) | *Tablet* | $ 0.027 | $ 0.027 | $ 0.069 | $ 0.120 | $ 0.027 |
| Diazepam (5 mg) | *Tablet* | $ - | $ - | $ - | $ 0.235 | $ - |
| Biperiden (2 mg) | *Tablet* | $ - | $ - | $ 0.040 | $ - | $ - |
|  |  |  |  |  |  |  |
| **Other information** |  |  |  |  |  |  |
| Average wage rate | *Month* | $ 25.02 | $ 60.61 | $ 90.42 | $ 120.46 | $ 14.28 |
|  |  |  |  |  |  |  |

Notes:

For all countries other than South Africa, unit costs of health services are based on WHO-CHOICE estimates, updated to the year 2015 (<https://www.who.int/choice/country/country_specific/en/>) and medication prices are taken from the International Medical Products Price Guide (<http://mshpriceguide.org/en/home/>). Derived values were shared and checked with local team investigators and agreed to be used as the basis for service costing. For South Africa, unit costs of health services are taken from the Department of Health’s Uniform patient fee schedule, and drug prices are taken from the Medicine Price Registry Database.
