# Supplementary material for: Health service costs and their association with functional impairment among adults receiving integrated mental health care in five low- and middle-income countries: the PRIME cohort study
Source: Health Policy Plan. 2020 Mar 9;35(5):567–76. doi: 10.1093/heapol/czz182 (PMC11318687; doi:10.1093/heapol/czz182)
Supplement: czz182_Supplementary_Data [file heapol_35_5_567_s1.zip › czz182-Suppl_Data/PRIME Cohort study of service costs (HPP tables 1-3).docx]

**Table 1 Sociodemographic characteristics of the PRIME cohort participants, 2015-2017.**

|  | Ethiopia (N=540) | |  | India (N=483)^b^ | |  | Nepal (N=433) | |  | South Africa (N=245) | |  | Uganda (N=295) | |
| --- | --- | --- | --- | --- | --- | --- | --- | --- | --- | --- | --- | --- | --- | --- |
|  | N | % |  | N | % |  | N | % |  | N | % |  | N | % |
| Sex |  |  |  |  |  |  |  |  |  |  |  |  |  |  |
| Male | 327 | 60.6 |  | 315 | 65.2 |  | 237 | 54.7 |  | 61 | 24.9 |  | 132 | 44.8 |
| Female | 213 | 39.4 |  | 168 | 34.8 |  | 196 | 45.3 |  | 184 | 75.1 |  | 163 | 55.2 |
| Age |  |  |  |  |  |  |  |  |  |  |  |  |  |  |
| 16-25 years | 175 | 32.4 |  | 88 | 18.2 |  | 40 | 9.2 |  | 28 | 11.4 |  | 124 | 42.0 |
| 26-35 years | 148 | 27.4 |  | 135 | 28.0 |  | 116 | 26.8 |  | 58 | 23.7 |  | 88 | 29.8 |
| 36-50 years | 155 | 28.7 |  | 185 | 38.3 |  | 172 | 39.7 |  | 70 | 28.6 |  | 65 | 22.0 |
| 51 years or more | 62 | 11.5 |  | 75 | 15.5 |  | 105 | 24.3 |  | 89 | 36.3 |  | 18 | 6.1 |
| Marital status |  |  |  |  |  |  |  |  |  |  |  |  |  |  |
| No partner | 307 | 56.9 |  | 42 | 8.7 |  | 78 | 18.0 |  | 134 | 54.7 |  | 213 | 72.2 |
| Has a partner | 233 | 43.1 |  | 441 | 91.3 |  | 355 | 82.0 |  | 111 | 45.3 |  | 82 | 27.8 |
| Educational level |  |  |  |  |  |  |  |  |  |  |  |  |  |  |
| Uneducated/illiterate | 303 | 56.3 |  | 140 | 29.0 |  | 105 | 24.3 |  | 9 | 3.7 |  | 60 | 20.3 |
| Non-formal/less than primary | 101 | 18.8 |  | 115 | 23.8 |  | 110 | 25.4 |  | 69 | 28.2 |  | 172 | 58.3 |
| Primary school and above | 134 | 24.9 |  | 228 | 47.2 |  | 218 | 50.3 |  | 167 | 68.2 |  | 63 | 21.4 |
| Employment ^a^ |  |  |  |  |  |  |  |  |  |  |  |  |  |  |
| Unemployed/not salaried | 2 | 6.9 |  | 152 | 31.5 |  | 217 | 50.1 |  | 185 | 75.5 |  | 196 | 66.4 |
| Employed | 27 | 93.1 |  | 331 | 68.5 |  | 216 | 49.9 |  | 60 | 24.5 |  | 99 | 33.6 |
| Food insecurity ^a^ |  |  |  |  |  |  |  |  |  |  |  |  |  |  |
| No | 29 | 96.7 |  | 460 | 95.4 |  | 102 | 23.6 |  | 137 | 55.9 |  | 233 | 79.0 |
| Yes | 1 | 3.3 |  | 22 | 4.6 |  | 331 | 76.4 |  | 108 | 44.1 |  | 62 | 21.0 |

^a^ In Ethiopia, baseline data collected only for AUD cohort; ^b^ Sociodemographic data missing for 19 cases

**Table 2 Baseline costs of health care, travel and time for PRIME cohort participants (US$, 2015)**

|  | | **Ethiopia** | | | **India** | | | **Nepal** | | | **South Africa** | | | **Uganda** | | |
| --- | --- | --- | --- | --- | --- | --- | --- | --- | --- | --- | --- | --- | --- | --- | --- | --- |
|  | | N | Mean | SD | N | Mean | SD | N | Mean | SD | N | Mean | SD | N | Mean | SD |
| Service cost, by type of care/service^1^ | | 540 | 5.72 | 21.08 | 502 | 3.00 | 9.06 | 433 | 10.17 | 29.95 | 245 | 23.01 | 41.22 | 295 | 6.70 | 10.86 |
| - Inpatient care | | 540 | 1.47 | 10.26 | 502 | 1.24 | 8.78 | 433 | 3.89 | 21.43 | 245 | 7.67 | 32.68 | 295 | 1.80 | 7.14 |
| - Outpatient care | | 540 | 4.25 | 17.86 | 502 | 1.35 | 1.67 | 433 | 4.49 | 20.12 | 245 | 13.37 | 19.08 | 295 | 3.25 | 7.83 |
| - - Mental health services | | 540 | 0.69 | 2.93 | 502 | 0.33 | 1.05 | 433 | 1.10 | 15.64 | 245 | 2.02 | 10.21 | 295 | 1.02 | 2.43 |
| - - General health services | | 540 | 1.95 | 11.57 | 502 | 0.85 | 1.13 | 433 | 1.62 | 5.87 | 245 | 10.02 | 12.34 | 295 | 0.95 | 3.05 |
| - - Indigenous / traditional services | | 540 | 1.61 | 13.01 | 502 | 0.17 | 0.62 | 433 | 1.27 | 11.14 | 245 | 1.33 | 6.08 | 295 | 1.29 | 6.87 |
| - Medication | | - | - | - | 502 | 0.41 | 0.57 | 433 | 1.79 | 2.56 | 245 | 1.97 | 7.46 | 295 | 1.64 | 2.11 |
|  | | | | | | | | | | | | | | | | |
| Travel time and costs^1^ | |  |  |  |  |  |  |  |  |  |  |  |  |  |  |  |
| - Accessing / waiting for services | | 540 | 2.27 | 5.20 | 502 | 0.98 | 1.33 | 433 | 1.84 | 4.37 | 245 | 12.63 | 14.90 | 295 | 0.98 | 1.64 |
| - Travel payments | | 540 | 11.16 | 52.31 | 502 | 0.59 | 1.04 | 433 | 0.64 | 2.62 | 245 | 1.18 | 4.24 | 295 | 1.74 | 4.53 |
|  | |  |  |  |  |  |  |  |  |  |  |  |  |  |  |  |
| Total out-of-pocket expenditure by households^1^ | | 540 | 15.78 | 61.64 | 502 | 2.26 | 6.96 | 433 | 10.43 | 30.59 | 245 | 6.68 | 13.12 | 295 | 5.63 | 10.17 |
| (travel costs, fees, medication) | |  |  |  |  |  |  |  |  |  |  |  |  |  |  |  |
|  | |  |  |  |  |  |  |  |  |  |  |  |  |  |  |  |
| **Service cost, by level of functional impairment** | | | | | | | | | | | | | | | | |
| - Lowest (<85^th^ percentile) | | 170 | 3.96 | 17.32 | 309 | 1.69 | 3.36 | 233 | 9.08 | 29.02 | 108 | 20.90 | 41.01 | 125 | 5.15 | 3.76 |
| - Higher (>=85^th^ percentile) | | 367 | 6.56 | 22.67 | 173 | 4.77 | 13.83 | 200 | 11.44 | 31.03 | 137 | 24.67 | 41.45 | 170 | 7.83 | 13.33 |
| *Difference (95% CI)* | *Higher impairment* | ***2.40 (0.13 to 4.67) **** | | | ***3.09 (0.76 to 5.42) ***** | | | *2.16 (-3.88 to 8.20)* | | | *6.60 (-3.54 to 16.74)* | | | ***2.36 (-0.09 to 4.82) ^m^*** | | |
|  | |  |  |  |  |  |  |  |  |  |  |  |  |  |  |  |
| **OOP expenditure, by level of functional impairment** | | | | | | | | | | | | | | | | |
| - Lowest (<85^th^ percentile) | | 170 | 14.24 | 77.07 | 309 | 1.33 | 2.86 | 233 | 9.03 | 29.36 | 108 | 3.91 | 12.07 | 125 | 4.05 | 4.61 |
| - Higher (>=85^th^ percentile) | | 367 | 16.52 | 53.43 | 173 | 3.41 | 10.21 | 200 | 12.05 | 31.96 | 137 | 6.50 | 13.94 | 170 | 6.79 | 12.69 |
| *Difference (95% CI)* | *Higher impairment* | *0.04 (-11.95 to 12.03)* | | | ***2.08 (0.37 to 3.79) **** | | | *2.78 (-2.93 to 8.50)* | | | *0.45 (-2.86 to 3.80)* | | | ***2.69 (0.38 to 5.01) **** | | |

^1^ All cost estimates relate to the three-month period leading up to baseline assessment.

^m^ marginal; * p<0.05; ** p<0.01

**Table 3 Health care costs and out-of-pocket payments for PRIME cohort participants at baseline, mid-line and end-line assessment (US$, 2015)**

|  | **Ethiopia** | | | **India** | | | **Nepal** | | | **South Africa** | | | **Uganda** | | |
| --- | --- | --- | --- | --- | --- | --- | --- | --- | --- | --- | --- | --- | --- | --- | --- |
|  | N | Mean | SD | N | Mean | SD | N | Mean | SD | N | Mean | SD | N | Mean | SD |
| **Total service cost in last 3 months** | | | | | | | | | | | | | | | |
| - Baseline | 540 | 5.72 | 21.08 | 502 | 3.00 | 9.06 | 433 | 10.17 | 29.95 | 245 | 23.01 | 41.22 | 295 | 6.70 | 10.86 |
| - Midline | 367 | 1.95 | 6.53 | 465 | 3.32 | 12.26 | 367 | 7.24 | 16.22 | 194 | 47.12 | 66.28 | 282 | 5.53 | 7.33 |
| - Endline | 446 | 1.65 | 6.95 | 433 | 5.18 | 19.44 | 370 | 11.19 | 35.41 | 183 | 17.36 | 30.53 | 259 | 3.59 | 5.21 |
| *Test of difference (coefficient, 95%CI)* | | | | | | | | | | | | | | | |
| - Midline versus Baseline | ***-4.08 (-5.62 to -2.55) ****** | | | *0.32 (-0.67 to 1.31)* | | | ***-3.16 (-5.73 to -0.60) ***** | | | ***26.25 (16.26 to 36.23) ****** | | | ***-1.15 (-2.32 to 0.03) ^m^*** | | |
| - Endline - Baseline | ***-3.72 (-5.09 to -2.34) ****** | | | ***2.14 (0.27 to 4.01) **** | | | *0.75 (-3.34 to 4.85)* | | | *-3.56 (-9.33 to 2.21)* | | | ***-3.03 (-4.11 to -1.95) ****** | | |
|  | | | | | | | | | | | | | | | |
| **Change in total service cost (endline – baseline)** | | | | | | | | | | | | | | | |
| - Functioning worsens ^a^ | 182 | -4.20 | 25.14 | 209 | 2.72 | 21.77 | 114 | 6.43 | 50.30 | 66 | -0.46 | 53.49 | 94 | -1.28 | 8.02 |
| - Functioning improves ^b^ | 259 | -2.64 | 15.12 | 203 | 2.04 | 21.34 | 256 | -2.24 | 41.95 | 117 | -4.87 | 36.52 | 164 | -3.86 | 13.45 |
| *Test of association (β, 95% CI)* | *-0.59 (-2.19 to 1.02)* | | | *0.01 (-3.90 to 3.92)* | | | ***-9.29 (-18.15 to -0.43) **** | | | ***-8.84 (-18.97 to 1.28) ^m^*** | | | *-0.49 (-1.88 to 0.90)* | | |
|  |  |  |  |  |  |  |  |  |  |  |  |  |  |  |  |
| **Total private, out-of-pocket cost in last 3 months** | | | | | | | | | | | | | | | |
| - Baseline | 540 | 15.78 | 61.66 | 502 | 2.26 | 6.96 | 433 | 10.43 | 30.59 | 245 | 6.68 | 13.12 | 295 | 5.63 | 10.17 |
| - Midline | 367 | 4.05 | 10.14 | 465 | 2.64 | 12.14 | 367 | 7.75 | 17.19 | 194 | 5.57 | 11.81 | 282 | 4.87 | 7.29 |
| - Endline | 446 | 3.26 | 6.96 | 433 | 4.73 | 19.25 | 370 | 11.65 | 36.81 | 183 | 4.96 | 9.17 | 259 | 3.95 | 6.09 |
| *Test of difference (coefficient, 95%CI)* | | | | | | | | | | | | | | | |
| - Midline - Baseline | ***-12.95 (-17.75 to -8.16) ****** | | | *0.39 (-0.57 to 1.36)* | | | ***-2.96 (-5.58 to -0.35) **** | | | *-0.24 (-2.12 to 1.65)* | | | *-0.79 (-1.93 to 0.35)* | | |
| - Endline - Baseline | ***-12.40 (-16.45 to -8.34) ****** | | | ***2.42 (0.52 to 4.32) **** | | | *0.93 (-3.30 to 5.16)* | | | *-1.02 (-2.83 to 0.78)* | | | ***-1.69 (-2.87 to -0.50) ***** | | |
|  | | | | | | | | | | | | | | | |
| **Change in out-of-pocket cost (endline – baseline)** | | | | | | | | | | | | | | | |
| - Functioning worsens ^a^ | 182 | -15.65 | 86.01 | 209 | 2.86 | 21.70 | 114 | 7.03 | 49.82 | 66 | 0.52 | 15.56 | 94 | -0.34 | 7.85 |
| - Functioning improves ^b^ | 259 | -9.94 | 43.51 | 203 | 2.13 | 19.26 | 256 | -2.24 | 43.67 | 117 | -1.85 | 15.07 | 164 | -2.45 | 13.30 |
| *Test of association (β, 95% CI)* | *-1.07 (-2.57 to 0.43)* | | | *-0.21 (-4.10 to 3.69)* | | | ***-9.50 (-18.18 to -0.81) ^*^*** | | | ***-2.96 (-6.08 to 0.17) ^m^*** | | | *-0.09 (-1.58 to 1.41)* | | |

^m^ marginal; * p<0.05; ** p<0.01; *** p<0.001; ^a^ defined as a follow-up WHODAS score equal to or greater than a WHODAS score at baseline; ^b^ defined as a follow-up WHODAS score smaller than a baseline WHODAS score.
